# Eye Movement Patterns in Natural Reading: A Comparison of Monolingual and Bilingual Reading of a Novel

**DOI:** 10.1371/journal.pone.0134008

**Published:** 2015-08-19

**Authors:** Uschi Cop, Denis Drieghe, Wouter Duyck

**Affiliations:** 1 Department of Experimental Psychology, University of Ghent, Ghent, Belgium; 2 School of Psychology, University of Southampton, Southampton, United Kingdom; Beijing Normal University, CHINA

## Abstract

**Introduction and Method:**

This paper presents a corpus of sentence level eye movement parameters for unbalanced bilingual first language (L1) and second-language (L2) reading and monolingual reading of a complete novel (56 000 words). We present important sentence-level basic eye movement parameters of both bilingual and monolingual natural reading extracted from this large data corpus.

**Results and Conclusion:**

Bilingual L2 reading patterns show longer sentence reading times (20%), more fixations (21%), shorter saccades (12%) and less word skipping (4.6%), than L1 reading patterns. Regression rates are the same for L1 and L2 reading. These results could indicate, analogous to a previous simulation with the E-Z reader model in the literature, that it is primarily the speeding up of lexical access that drives both L1 and L2 reading development. Bilingual L1 reading does not differ in any major way from monolingual reading. This contrasts with predictions made by the *weaker links account*, which predicts a bilingual disadvantage in language processing caused by divided exposure between languages.

## Introduction

By now, psycholinguistics has gained a good understanding of monolingual reading behavior. However, because of the increased globalization of our multicultural society, more and more people acquire, apart from their mother tongue (L1), one or more other languages (L2, L3…). It is now estimated that about half of the world’s population has some knowledge of more than one language, and can therefore considered to be bilingual, following the common Grosjean definition: “*bilinguals are those people who need and use two (or more) languages in their everyday lives*” [1]. In contrast, current models of eye movements during reading still focus exclusively on monolingual reading, so that we do not know in what way L2 sentence reading differs from L1 reading, or whether merely being a bilingual changes L1 reading.

In contrast to the monolingual domain, almost all studies of bilingual reading have focused on the word level. The few studies that do use sentence materials suggest that having a second language available influences the way the first language is processed [2, 3]. They do not however consider sentence-level reading parameters, as was done in the monolingual domain [4–6], but rather focus on the recognition of target words that are embedded in a sentence context [2, 3, 7–15]. The present study aims to address this gap by providing a systematic investigation of eye movements when bilinguals read in their native and second language. These data constitute the necessary constraints to generalize models of eye movement behavior to bilingual readers.

### Monolingual Eye Movements while Reading

When we read, our eyes move from one position to the next in order to identify and process visual word form information. This entails rapid jerk-like movements (saccades) and short periods of steadiness (fixations). Saccades are necessary to direct the gaze to a new location, bringing new information into the center of the visual field where acuity is best. During these saccades, no meaningful new visual information is gathered. They occur several times per second and typically move the eyes forward about 7–9 character spaces (for reviews:[16, 17]. Psycholinguists assume that eye movements during reading reflect language processing [18], with fixation durations as a marker of the ease of accessing the meaning of a word and integrating this into the current sentence. Because of the spatially accuracy and high temporal resolution of eye tracking, it allows us to dissociate early from late eye movement measures. In combination with other information, such as word length/frequency, this makes it possible to investigate the time course of the reading process. Additionally, reading processes in eye tracking are not confounded by task-related processes or strategies that other lab tasks (e.g. lexical decision or naming) entail. Hence, this method is considered to be the closest experimental parallel to the natural reading process.

During the last three decades, the development of monolingual theories on visual language comprehension has been heavily influenced by eye tracking research in reading. Rayner’s influential review article [16], now 15 years old, already discusses more than 550 articles investigating this topic (for a more recent review:[17]). Also, several corpus studies of eye movements were undertaken, and these data were used to provide an account of (monolingual) reading. The Potsdam Corpus [19, 20] contained eye movements of 222 subjects reading 144 constructed German sentences (1 138 words). The Dundee corpus [21], an English and French study in which 10 participants read 50 000 words in paragraphs, was used to investigate effects of parafoveal processing. Clearly, these corpora of eye movements provide a very rich and extended source of information about the mechanisms that underlie language processing in a more natural context and could serve as harvesting grounds for the development of comprehensive language models. For example, the Amherst Sentence Corpus [22] was used to develop the first version of the SWIFT model of saccade generation [23].

The E-Z reader model [22, 24–29] is the most cited model of monolingual eye movements. It is implicitly limited to native language or even monolingual reading behavior, and it is yet unknown how these mechanisms operate when bilinguals read in a second language, or how knowledge of a second language influences native language reading. However, it is interesting that the original E-Z reader model has been successfully accommodated to account for other reading patterns, such as those of older readers [30], children[31], or of non-alphabetic languages[32]. This illustrates that this model could be useful and relevant in future modeling efforts concerning bilingual eye movement patterns, and we will therefore align our analyses of bilingual reading behavior with the core assumptions and variables of this model.

The E-Z reader model assumes serial lexical processing. The completion of an early stage of lexical processing on word *n*, called the *familiarity check*, is the ‘trigger’ that causes the oculo-motor system to begin the programming of a saccade directed towards the next word *n+1*. The subsequent completion of a second stage of lexical processing on word *n*, called the *completion of lexical access*, causes attention to shift from word *n* to word *n+1*. Thus, the programming of saccades is decoupled from the shifting of attention, which is allocated serially to only a single word at a time [22]. Because attention shifts are faster than the programming of a saccade[30], the lexical processing of word *n+1* usually begins when the eyes are still fixated on word *n*. This feature of the model allows parafoveal processing of upcoming words. Following similar reasoning, the model predicts that parafoveal words, which are processed fast enough, might be skipped.

The model assumes that word length and frequency are important lexical variables that have a large effect on the eye movements, because these variables define the duration of the familiarity check [33, 34]. Consequently, they determine fixation duration, fixation count, rightward saccade length, skipping and regression rates. These will also be the core variables that will be assessed in the present paper.

### Research on Bilingualism

Most bilingual language research has focused on the question of how the bilingual lexicon is organized. Do people have separate representational systems for lexical items of different languages or is there one integrated lexicon? Although intuitively the most straightforward option might be to have a separate lexicon for each language, and although bilinguals can use one of their languages without the constant intrusion of the other language [35], the large majority of experimental evidence shows that bilinguals have one integrated lexicon containing representations of all words belonging to both languages and that this lexicon is accessed language independently [36]. Evidence for this idea is mainly provided by research on cross-lingual interactions, in which it is typically shown that words with some overlap across languages are processed differently than control words, even during unilingual processing. Most often these overlapping words are cognates presented in isolation [37–50]. Cognates are words that are translation equivalents but also show some degree of form overlap (e.g. Dutch-English *appel; apple*). Research shows that bilinguals identify cognates faster than control words in a lexical decision task (e.g. 35,36), a translation priming task (e.g. 32,33) and a progressive demasking task (e.g. 30). This is the case when participants perform the task in their L2 (e.g. 23–27) and in their L1 (e.g. 28), although the effect is usually larger for L2 [43]. These cross-lingual interaction effects are also found when a target word is embedded in a sentence context [51–53]. This means that a unilingual sentence context does not restrict lexical access to only the target language. In this way these studies provide evidence for a language non-selective view on bilingual language processing. For an overview of evidence for cross-lingual activation and an integrated bilingual lexicon see Brysbaert and Duyck’s [54] or Van Hell and Tanner’s [55] overview.

All of the bilingual research discussed in the previous paragraphs used an alternative method to eye movement recording, such as word naming, categorization tasks or lexical decision tasks to examine lexical processing. Although these tasks have their merits for investigating word recognition in isolation, there also have some limitations, besides those mentioned in the previous section, that make these methods suboptimal for investigating lexical access in natural reading. In natural reading, word processing is influenced by the sentence context and parafoveal stimuli [56]. This suggests that words are processed gradually across time and across multiple fixations. Also, during reading of text lexical access takes place while other cognitive processing is going on. Kuperman, Drieghe, Keuleers and Brysbaert indeed show that only 5–17% of the variance in gaze durations on target words embedded in sentences is explained by lexical decision times in isolation after partialling out the effects of word frequency and word length [57]. This illustrates that the two approaches are indeed distinguishable and measure, to a large extent, different language processes, making both approaches indispensable to research into language processes. Given that only eye tracking assesses reading behavior as it occurs in natural language processing, it is important not to rely solely on artificial word processing paradigms such as lexical decision tasks for the development of models of reading but to complement them with natural reading tasks.

As mentioned above, monolingual theories on visual word recognition have advanced much through eye tracking studies. In the bilingual domain, most eye tracking studies examined eye movements to detect cross-lingual activation in bilingual reading [2, 3, 7–10, 58, 59]. Other eye-tracking studies have focused on syntactic processing [11, 12], the effect of semantic constraint [14], frequency effects [13, 60] or inter-word spacing effects [15] in bilingual visual word recognition.

Most studies that tracked eye movements in bilinguals examined the fixations directed towards the embedded target words, or some other critical target area, without taking into account changes in global eye movement behavior that L2 reading might entail [2, 3, 7–14]. Although Titone et al. [3] and Altarriba et al. [14] do provide some basic word-level eye movement measures for paragraph reading as a measure of reading proficiency, Whitford and Titone [60] were the first to analyze bilingual eye movements to all words, not just target words, in bilingual paragraph reading. These data are still presented on a word level. To our knowledge there is only one bilingual eye tracking study, Winskel et al. [15], that provides sentence level reading measures for bilingual sentence reading. They give the sentence reading time and fixation count for 36 English-Thai bilinguals reading 72 Thai and English spaced and un-spaced sentences. See Van Assche, Duyck and Hartsuiker [61] and Dussias [62] for an overview of the use of eye movements in bilingual sentence processing research.

### Theories about Bilingual Word Recognition

The most cited, and the only implemented, model of bilingual visual word recognition is the Bilingual Interactive Activation plus (BIA+) model [36]. This model is an adaptation of the interactive activation model of word recognition [63]. The main differences are the inclusion of lexical representations of two languages, and a distinction between a word identification system and a task/decision system. The BIA+ states that during bilingual reading there is parallel, language independent activation of lexical representations in an integrated lexicon. Language nodes that represent language membership are included in the model, but they cannot tune word recognition towards a single language via top-down activation. This architecture implies that for every word bilingual readers encounter all lexical candidates from all known languages are activated to some extent. Evidence for this model is generated by studies supporting cross-lingual interactions (see previous paragraph for references).

A limitation of the BIA+ model [36], similar to the monolingual interactive activation model [63] is that it is tailored to isolated word recognition, and not to sentence reading. The authors do assume effects of sentence context and non-linguistic information on word recognition but the exact nature of these interactions are not specified. This means that a model of bilingual eye movements, such as the E-Z reader model, is not yet available, as there is also no sentence reading data to base it upon.

The *weaker links account* [13, 64], sparked by small but consistent production disadvantages exhibited by bilinguals compared to monolinguals [65–67, 13, 68], has recently gained popularity in the literature. Like the BIA+ model [36], it assumes an integrated bilingual lexicon. According to this *frequency-lag* account, bilinguals will have about double the amount of lexical items in their lexicon as monolinguals and will necessarily divide the frequency of use of these words between languages [64]. Considering the lexical quality hypothesis [69,70], which states that increased word practice results in better precision of the corresponding lexical representations, it is plausible that bilingual representations will be of lower precision than those of monolinguals. Indeed, Gollan, Montoya, Cera and Sandoval predict that weaker links between word form and representations for bilinguals should result in slower lexical access during language comprehension, either while accessing L1 or L2, compared to monolinguals [64]. Effects might be smaller than in production because the processes needed for language production are less practiced, more difficult and involve more levels of processing for which frequency is important [13]. In the comprehension domain, it was indeed found that bilinguals show slower L1 lexical decision times than monolinguals do [71, 72].

A core assumption at the heart of the weaker links account is that total language exposure is equal for all people. While this maybe the case for bilinguals who are exposed to two languages from birth, it is definitely not true for all groups of bilinguals. The authors that constructed the weaker links account used mostly early Spanish English bilinguals [13, 64, 67]. A population of unbalanced bilinguals usually acquires a 2^nd^ language in a classroom context, thus increasing their total vocabulary and language exposure, not per se decreasing their L1 exposure. On top of that, the words of their mother tongue will have been fully lexically entrenched before they start learning their second language. This means that for late learners of an L2, the lexical entrenchment for L1 words might be equally strong as the lexical entrenchment for the words of a monolingual.

### This Study

The current paper provides the first comprehensive description of bilingual (L1 and L2) and monolingual reading on a sentence level by gathering a corpus of eye movement data while participants read an entire novel. Within this single data set a wide range of phenomena can be studied in an ecologically valid context and benchmark parameters of bilingual L1 and L2 natural sentence reading can be extracted. This corpus enables the examination of global changes in eye movement pattern, clarifying localized measures associated with the identification of specific words embedded within a sentence. To be more specific, if our analysis for instance shows that average saccade length is typically reduced in L2 reading compared to L1 reading, this would influence factors that are normally associated with the lexical processing of a specific word (e.g. word skipping, number of fixations) even though these patterns would only reflect global adjustments to reading in L2 and not just the lexical processing of the currently fixated word. Ultimately, these results will promote the development of models and theories on bilingual language processing in L1 and L2.

The aim of this paper is twofold. First, we will compare eye movement patterns of bilinguals reading in L1 and L2. We will use a within-subjects design. In this way, reading language is not confounded with inter-individual differences such as motivation or intelligence. A direct comparison of individuals’ reading performance across languages is rather challenging. We discuss this issue in the section ‘Analytic Techniques for Cross-Language Comparison’.Second, we want to investigate whether merely being a bilingual changes native language reading, by comparing bilingual L1 (Dutch) with monolingual L1 (English) reading of cross-lingually matched sentences (between-subjects).

#### Predictions L1 vs. L2 Reading

As discussed, the weaker links account predicts a disadvantage for the least frequently used language dependent on the relative exposure of L1 and L2, caused by weaker links between L2 word forms and representations [64]. Although some of the studies described above, for example Whitford and Titone [60], observed longer gaze durations and longer sentence reading times on embedded target words in L2 sentences, no study so far has compared basic sentence parameters for L1 and L2 reading.

We can draw a parallel between the sentence reading pattern of children and the expected sentence reading pattern for unbalanced bilinguals reading in L2. Unbalanced bilinguals are also developing, although for the second time, reading skills. For bilinguals, the first stages of letter recognition should already have been automatized, so on a quantitative level, we expect that the size of the difference between L1 and L2 bilingual reading measures should be somewhat smaller than the size of the difference between adults’ and children’s reading measures.

As children acquire reading skills and gain language proficiency, sentence reading times and fixation durations get shorter, saccade length gets longer, and fewer fixations, regressions and refixations are made [4–6, 73–78]. Interestingly, these are strictly quantitative, rather than qualitative differences. This robust evolution is most likely due to a speeding of the lexical identification of the individual words [73] not by oculomotor development [4, 5, 76] So, although children are slower, they do not need more time than adults do to take up the necessary information from the page. Reichle et al. [31] confirmed this using a simulation of the eye movement data of children using the E-Z reader model [22]. The full eye movement pattern of children was simulated by lowering the default rate of lexical processing compared to adults. This supports the fact that the tuning of the oculomotor system is not the main element that drives the development of eye movement behavior in children [31].

Rayner, Reichle, Stroud, Williams and Pollatsek described a “risky reading strategy” for older readers as a compensation mechanism for slower lexical access. Older people fixate longer on individual words in a sentence and make more regressions in the text, but also that they skip more words and move their eyes with bigger saccades over the text [30].

In summary, given lower language proficiency for L2, we predict a “child-like” eye movement pattern for bilinguals reading in their L2 vs. their L1. This is compatible with the weaker links hypothesis, which also assumes effects of lower L2 practice. This disadvantage should be more pronounced in readers who score lower on L2 proficiency. We predict more and longer fixations per sentence, a smaller rightward saccade length, a lower skipping rate and a higher regression rate for L2, but we keep in mind that this pattern might be compensated by strategically adjusting the skipping rates and saccade length, as Rayner observed for older readers [30].

#### Predictions Monolingual vs. Bilingual Reading

For bilinguals, reading experience is supposedly spread across two different languages, L1 and L2 [64]. This implies lower absolute exposure to each language, which could result in slower lexical access and thus word recognition [71, 72] and reading for bilinguals compared to monolinguals. We expect that the weaker links account does not apply to late bilinguals, per se, because these participants might have experienced larger language exposure in general than monolinguals have and because lexical entrenchment of L1 words is in an advanced stage before learning an L2.

Although Gollan et al.’s eye tracking study [13] does explicitly compare English monolinguals with balanced Spanish-English bilinguals on an English reading task, their bilingual group scored worse on the objective English proficiency measure than their monolingual group did [13]. Bilinguals accordingly showed longer gaze duration and lower skipping rates for the target words than monolinguals did. It is thus unclear whether this difference is a necessary and intrinsic consequence of bilingualism or rather whether it is driven by proficiency.

In our study, we excluded language proficiency as a possible confounding variable by matching our bilingual’s L1 proficiency to our monolingual’s language proficiency. Note, that similar proficiency scores would already imply that the lexical entrenchment of the bilinguals’ L1 is on the same level as the lexical entrenchment of the monolinguals.

In conclusion, the weaker links account predicts slower sentence reading times, more and longer fixations per sentence, a smaller saccade length, lower skipping rates and higher regression rates, for bilinguals reading in L1 than for monolinguals. These differences will be subtler than the differences between the bilingual L1 and bilingual L2 reading pattern, because the L1 proficiency is the same for both groups. When we assume similar L1 lexical entrenchment for unbalanced bilinguals, we would expect a similar global eye movement pattern for monolinguals and bilinguals reading in their L1.

## Method

The ethical committee of the University of Ghent approved the experimental procedure (nr. 2011/44). Participants signed an informed consent form prior to starting the experimental procedure. A summary of this method is included in Cop, Keuleers, Drieghe, and Duyck [79], because that study presented other analyses of the same eye-tracking corpus data, focusing specifically on word-level frequency effects, rather than the broad sentence-level differences investigated in the present study.

### Participants

Nineteen unbalanced Dutch (L1)–English (L2) bilingual Ghent University and fourteen English monolingual Southampton University undergraduates participated either for course credit or monetary compensation. Bilingual and monolingual participants were matched on age and education level. The average age was 21.2 years for bilinguals [range: 18–24; sd = 2.2] and 21.8 years for monolinguals [range: 18–36, sd = 5.6]. All of the participants were enrolled in a bachelor or master program of psychology. In the monolingual group, 6 males and 7 females participated. In the bilingual group, 2 males and 17 females participated.

Participants had normal or corrected-to-normal vision. None of the participants reported to have any language and/or reading impairments.

The bilinguals had a relatively late age of acquisition for L2: The mean age of acquisition was eleven years [range: 5–14, sd = 2.46]. All participants completed a battery of language proficiency tests, including a spelling test, the LexTALE [80] and a lexical decision task (for results see Table 1). For the bilinguals, a self-report language questionnaire was added. This contained questions about language switching frequency/skill, age of L2 acquisition, frequency of L2 use and reading/auditory comprehension/speaking skills in L1 and L2. All of the bilinguals report that they can carry on a conversation, read and comprehend instructions, sometimes read articles, books, watch TV shows and listen to music in English (their L2). The bilinguals report that they use their L2 on average 3.6 days a week (range: 1–7 days). About half of the bilinguals also report that they sometimes think or talk to themselves in English (for a detailed summary, see Tables A and B in S1 File) Due to the lack of a standardized cross lingual spelling test, we tested the English spelling with the spelling list card of the WRAT 4 [81] and the Dutch spelling with the GLETSCHR [82]. The LexTALE (Lexical Test for Advanced Learners of English) is an unspeeded lexical decision task, which is an indicator of language proficiency for intermediate to highly proficient language users, validated for English, Dutch and German [80]. Two bilinguals were classified as lower intermediate L2 language users (50%-60%), ten bilinguals were classified as upper intermediate L2 language users (60%-80%), seven bilinguals scored as advanced L2 language users (80%-100%) according to the LexTALE norms reported by Lemhöfer and Broersma [80]. A classical speeded lexical decision task was also administered in Dutch and English for the bilinguals, in English for the monolinguals. We calculated a composite proficiency score by averaging the score on the spelling test, the score on the LexTALE and the adjusted score of the L2 lexical decision task. Table 1 shows, mean accuracy for the spelling tests and LexTALE, lexical decision word accuracy corrected for false alarms, and the composite proficiency score.

**Table 1:** Average percentage scores [standard deviations] on the LexTALE, Spelling test and Lexical Decision task for the bilingual and monolingual group. T-values [degrees of freedom] of t-tests in the last 2 columns.

	Monolinguals	Bilinguals L1	Bilinguals L2	t-value L1-L2	t-value L1-mono
LexTALE score (%)	91.07 [8.92]	92.43 [6.34]	75.63 [12.87]	7.59 [18] ***	0.49 [22.3]
Spelling score (%)	80.78% [7.26]	83.16 [7.80]	69.92 [8.74]	8.15 [18] ***	0.99 [29.3]
Lexical Decision score (%)	77.89 [12.01]	80.47 [5.45]	56.75 [11.01]	9.87 [18] ***	0.67 [17.1]
Composite Proficiency Score (%)	83.25 [8.30]	85.54 [4.68]	67.81 [9.72]	11.78 [18] ***	0.93 [19.1]

* p<0.05

** p<0.01

*** p<0.001

Most important, the Dutch (L1) proficiency of the bilinguals was matched with the English proficiency of the monolinguals (See column 5 in Table 1), indicating that both groups were equally proficient in their first language. The English (L2) proficiency is clearly lower than the Dutch (L1) proficiency (see column 4 in Table 1).

### Materials

The participants read the novel “The mysterious affair at Styles” by Agatha Christie (Title in Dutch: “De zaak Styles”). This novel was selected out of a pool of books that were available in a multitude of different languages (for possible future replication in other languages) and which did not have any copyright issues. All of these books were selected from the Gutenberg collection that is freely available on the Internet. We selected the novels that could be read in four hours. The remaining books were inspected for difficulty, indicated by the frequency distribution of the words that the book contained. The Kullback–Leibler divergence [83] was used to select the novel whose word frequency distribution was the most similar to the one in natural language use (according to the subtlex database). This novel also had one of the lowest number of hapax words (words that occur only once in the subtlex database) of the selected books.

Table 2 shows a summary of the characteristics of the Dutch and English version of the novel. The difference in number of words per sentence and average word length illustrates that English is a denser language than Dutch. Although the differences in absolute values were very small, paired t-tests still yielded significant differences between the two languages concerning number of words per sentence and average word length, because of the extremely big corpus size (*n* = 5 212). The difference between average content word frequencies was not significant.

**Table 2:** Summary of the characteristics of the translation equivalent sentences and the restricted set of sentences matched on information density (averages of Word Length, Number of Words per sentence, Number of Characters per sentence, Number of Content words per sentence, Word Frequency and Content word frequency) across languages.

Descriptive parameters	Translation equivalent sentences			Restricted set of sentences		
	Dutch	English	T value	Dutch	English	T value
Number of Words	55 596	51 594	-	1 628	1 628	-
Number of Sentences	4 804	4 804	-	210	210	-
Number of Words per Sentence	11.52 [8.89]	10.73 [8.10]	5.06***	7.53 [6.65]	7.53 [6.65]	-
Number of Characters per Sentence	51.76 [41.27]	43.28 [34.25]	12.40***	32.62 [28.76]	31.46 [27.85]	6.48***
Number of Content Words per Sentence	5.87 [4.58]	5.33 [4.06]	6.86***	3.68 [3.34]	3.76 [3.46]	-1.19 (p = 0.24)
Average Word Frequency	4.49 [0.60]	4.57 [0.59]	-6.86***	4.29 [0.88]	4.37 [0.87]	-3.23**
Average Content Word Frequency	3.84 [0.75]	3.85 [0.76]	-0.40 (p = 0.69)	3.89 [0.85]	3.86 [0.84]	0.86 (p = 0.39)
Average Word Length	4.52 [1.04]	4.18 [0.97]	16.93***	4.54 [1.42]	4.52 [1.42]	1.33 (p = 0.19)

* p<0.05

** p<0.01

*** p<0.001

### Apparatus

The bilingual eye movement data were recorded with a tower-mounted EyeLink 1000 system (SR-Research, Canada) with a sampling rate of 1 kHz. A chinrest was used to reduce head movements. Monolingual eye movement data were acquired with the same system that was desktop mounted. Reading was always binocular, but eye movements were recorded only from the right eye. For the bilingual participants, sentences were presented on a 22 inch Philips 202P70 CRT-monitor and for the monolingual participants, sentences were presented on a 21 inch g225f view Sonic graphics series monitor. Text was presented in black 14 point Courier New font on a light grey background. The lines were triple spaced and 3 characters subtended 1 degree of visual angle or 30 pixels. Text appeared in paragraphs on the screen. A maximum of 145 words, spread over a maximum of 10 lines, was presented on one screen. During the presentation of the novel, the room was dimly illuminated.

### Procedure

Participants read the entire novel in four sessions of an hour and a half. One bilingual participant read only the first half of the novel in English in two sessions. In the first session, every participant read chapter 1 to 4. In the second session chapters 5 to 7, in the third session chapters 8 to 10 and in the fourth session chapter 11 to 13 were read. The bilinguals read half of the novel in Dutch, the other half in English. The order was counterbalanced. The monolinguals read the entire novel in English. Every bilingual and monolingual participant completed a number of language proficiency tests. The results of these proficiency measures can be found in Table 1.

The participants were instructed to read the novel silently while the eye tracker recorded their eye movements. It was stressed that they should move their head and body as little as possible while they were reading. The participants were informed that there would be a break after each chapter and that in that pause they would be presented with multiple-choice questions about the contents of the book. This was done to ensure that participants understood what they were reading and paid attention throughout the session. The number of questions per chapter was relative to the amount of text in that chapter.

The text of the novel appeared on the screen in paragraphs. When the participant finished reading the sentences on one screen, they were able to press the appropriate button on a control pad to move to the next part of the novel.

Before starting the practice trials, a nine-point calibration was executed. The participants were presented with three practice trials where the first part of another story was presented on the screen. After these trials, the participants were asked two multiple-choice questions about the content of the practice story. This part was intended to familiarize participants with the reading of text on a screen and the nature and difficulty of the questions. Before the participant started reading the first chapter another nine-point calibration was done. After this, the calibration was done every 10 minutes, or more frequently when the experiment leader deemed necessary.

## Results

As described above, we analyzed the eye movement data at the sentence level. Data collection contained 5 212 data points or sentences per subject. Fixations shorter than 100ms were excluded from analyses. 243 (4.9%) unusual sentences were removed because they contained more than 35 words, had an average word length of more than 7.4 characters or had an average content word frequency lower than 1.56. This left us with 4 969 sentences per subject on average.

The bilinguals scored 81% [sd = 13.36] on the L1 multiple-choice questions and 79% [sd = 12.54] on the L2 multiple-choice questions. A paired t-test did not yield a significant difference between these two (t = 0.275, df = 17, p = 0.787). The monolinguals scored on average 78% [sd = 9.46]. A t-test did not yield a significant difference between the bilingual L1 and the monolingual comprehension scores (t = 0.675, df = 29. 79, p = 0.505). See S2 File for the questions and multiple-choice answers.

### Analytic Techniques for Cross-Language Comparison

Following our rationale, two comparisons are essential for this paper. The first one is the within-subject comparison of the bilingual L1 and L2 reading data to explore the influence of “Language” (L1 or L2); the second one is the comparison between bilingual L1 and monolingual reading in order to assess the possible effects of being a bilingual. Both comparisons imply by definition the need to directly compare reading behavior across two different languages. There might be inherent differences between languages relating to formal characteristics, information density and difficulty. This necessitates matching for inherent language differences that may influence basic reading characteristics. We tested Dutch-English bilinguals reading a novel in both Dutch and English. Dutch is the closest major language relative to English, so that this language pair is the best-suited combination starting from the dominant language in the reading literature (English).

First, there is a need for matching the materials on semantic content. We manually checked each sentence for translation equivalence. The sentences that did not match this criterion, and thus had slight semantic differences across languages, were excluded from all of the following analyses. 4 764 sentences per subject were retained for analysis (3.99% of Dutch and 3.95% of English sentences were excluded). The sentences were numbered pairwise and this “sentence identity number” will be used in the analysis.

Second, information density is an indication of the amount of syllables needed to convey a certain semantic content [84]. As we can see in Table 2, there are significant differences between measures of information density (average word length and number of words per sentence) for the two texts in the different languages. By including these factors as fixed effects in our linear mixed model, we made sure that the significance of the other fixed effects in the model is not affected by these differences. To be even more conservative, we created a more restricted data set by matching the sentences pairwise on average word length (threshold = 0.2 characters per sentence) and number of words per sentence (exactly matched) to equalize information density for each translation equivalent English-Dutch sentence pair. After this, text difficulty, as measured by the mean frequency of the content words, was still matched across languages. Only 4.2% of the sentences were retained in this selected dataset. This selection still contained 210 sentences per subject (for a summary of the lexical variables for the matched material set see Table 2). We report the results for this restricted, optimally matched data set, extracted from the natural reading corpus data.

### Model Fitting

For analysis, we selected the dependent variables that are well captured by models of reading such as the E-Z reader model. For both comparisons, the dependent measures under investigation are: a) sentence reading time including fixations and re-fixations, b) total number of fixations that landed in one sentence, c) the average fixation duration of the fixations that landed in that sentence, d) the average rightward saccade length per sentence, e) the probability of making an inter-word regression towards or within a certain sentence and f) the probability of first pass skipping.

Our data corpus was analyzed with linear mixed effects models with the lme4 (version 1.1–7) and lmertest (version 2.0–20) package of R (version 3.0.2) [85], because a multilevel design is the best way to statistically control for a range of predictors that in this experiment we could not or did not want to manipulate.

For the first within-subject comparison of the bilingual L1 vs. L2 reading data, the same fixed effects model was fitted for every eye movement measure. The fixed factors were language (L1 or L2), number of words per sentence (continuous), average word length per sentence (continuous), average frequency of the content words per sentence (continuous) and L2 proficiency (continuous). This last variable is the composite proficiency score presented in Table 1. Note that this variable represents something different for the two language conditions. For the L2 condition this is the language they are reading in. For the L1 condition it is their proficiency in a second language that they do not use in this condition. For the content word frequency, the subtitle word frequency measures[86, 87] of the content words in a particular sentence were log transformed to normalize their distribution. All continuous predictors were centered. The absolute value of the maximum correlations among main effects was under 0.51 for all eye movement measures (<0.506 for Saccade length, <0.156 for fixation count, <0.167 for fixation duration, <0.249 for dwell time, <0.386 for regressions, <0.245 for skips).

In a first step, we fitted a “complete” model. The fixed part of the model contained all main effects and interactions (up to 5-way) and the random part contained two random clusters: one for subject (the participant ID-number) and one for sentence (the sentence ID-number). After fitting this first model, we excluded the terms one by one, starting with the factor that contributed the least to the fit. By model comparisons, we decided when we arrived at the best possible fit. Then we added random slopes one by one. When they contributed to the fit, we included the slope in the model. We choose to test addition of every possible random slope, and strive for a maximal random structure [88]. We added, in this order, language as a random slope for each sentence and language, word length, word frequency and number of words as random slope per subject. For the count variable and the binomial variables (fixation count, skipping rate, regression rate) we report the p-values for the significant effects. For the continuous variables (sentence reading times, average fixation duration and saccade length), we obtained the p-values by computing the F-Test with Kenward-Roger adjusted degrees of freedom [89] for our fixed effects in the final models.

For the second important (between-subject) comparison between the bilingual L1 and monolingual L1 reading, the same model was fitted for every eye movement measure. Here, the fixed factors were bilingualism (Bilingual or Monolingual), number of words (continuous), average word length (continuous), average frequency of the content words (continuous) and L1 proficiency (continuous). This last variable is the composite proficiency score presented in Table 1. Note that for both the bilinguals and the monolinguals this is the language they are reading in. The frequency measure was computed the same way as in the previous comparison. The process of top-down fitting of fixed effects and bottom-up fitting of the random slopes was identical to the process in the first comparison. Again, a maximum random structure was aspired but this time we added, in this order, bilingualism as a random slope for each sentence and word length, word frequency and number of words as random slope per subject. Again, the p-values for the continuous variables were calculated with the F-test with Kenward-Rogers adjusted degrees of freedom [89].

### Bilingual L1 vs. Bilingual L2 Reading

#### Sentence Reading Time

Sentence reading times that differed more than 3 standard deviations from the general mean reveal unusual distraction and were therefore excluded from the analysis (5.02%). Sentence reading times were log transformed as suggested by the Box-Cox method [90] to obtain a more normal distribution and then analyzed with the linear mixed model described above.

**Fig 1:**
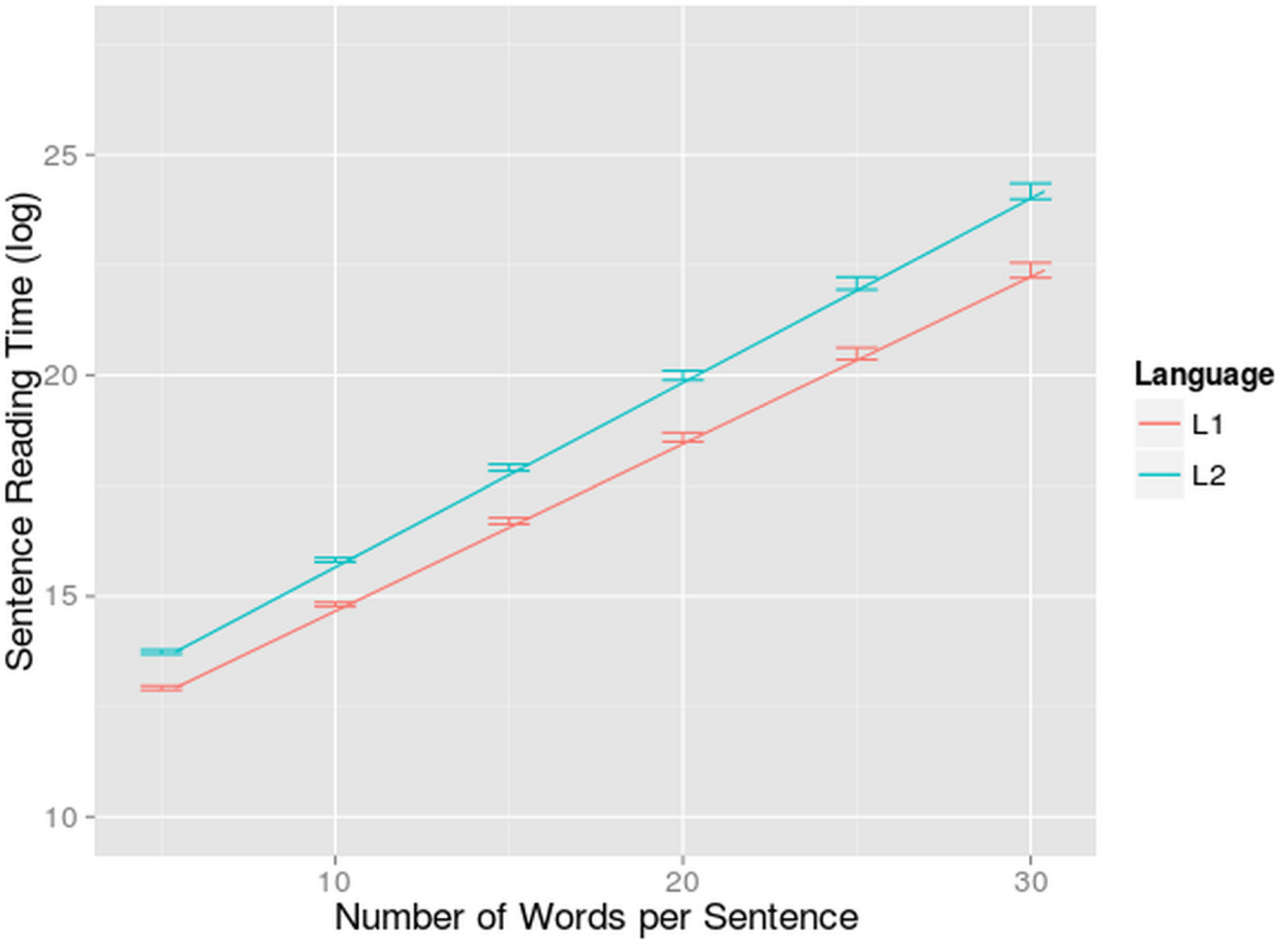
Interaction-effect of number of words and language on reading times. Sentence reading time (log-transformed on the y-axis) in function of number of words (on the x-axis) per sentence for bilinguals reading in L1 and L2. The standard errors are indicated by whiskers on the graph.

A main effect was found for language (F = 36.43, df = 24.70, p<0.001): the bilinguals were 17% slower to read a sentence in their L2 than in their L1 (1.52s compared to 1.27s), a rather large effect. This indicates that reading text in a less proficient second language produced an obvious disadvantage. This disadvantage was larger in longer sentences as shown by the interaction between language and number of words (F = 9.92, df = 207.54, p< 0.005). In other words, an extra word per sentence prolonged the reading time of an L2 reader more than the reading time of an L1 reader (Fig 1). This was probably caused by the fact that individual fixations were longer when reading in L2. This would accumulate into a longer reading time in longer sentences. Also, longer sentences often entail a higher syntactical complexity, which could come with a cost that is higher in L2 than in L1. When looking at the other dependent variables, it will become clear whether this explanation holds.

**Fig 2:**
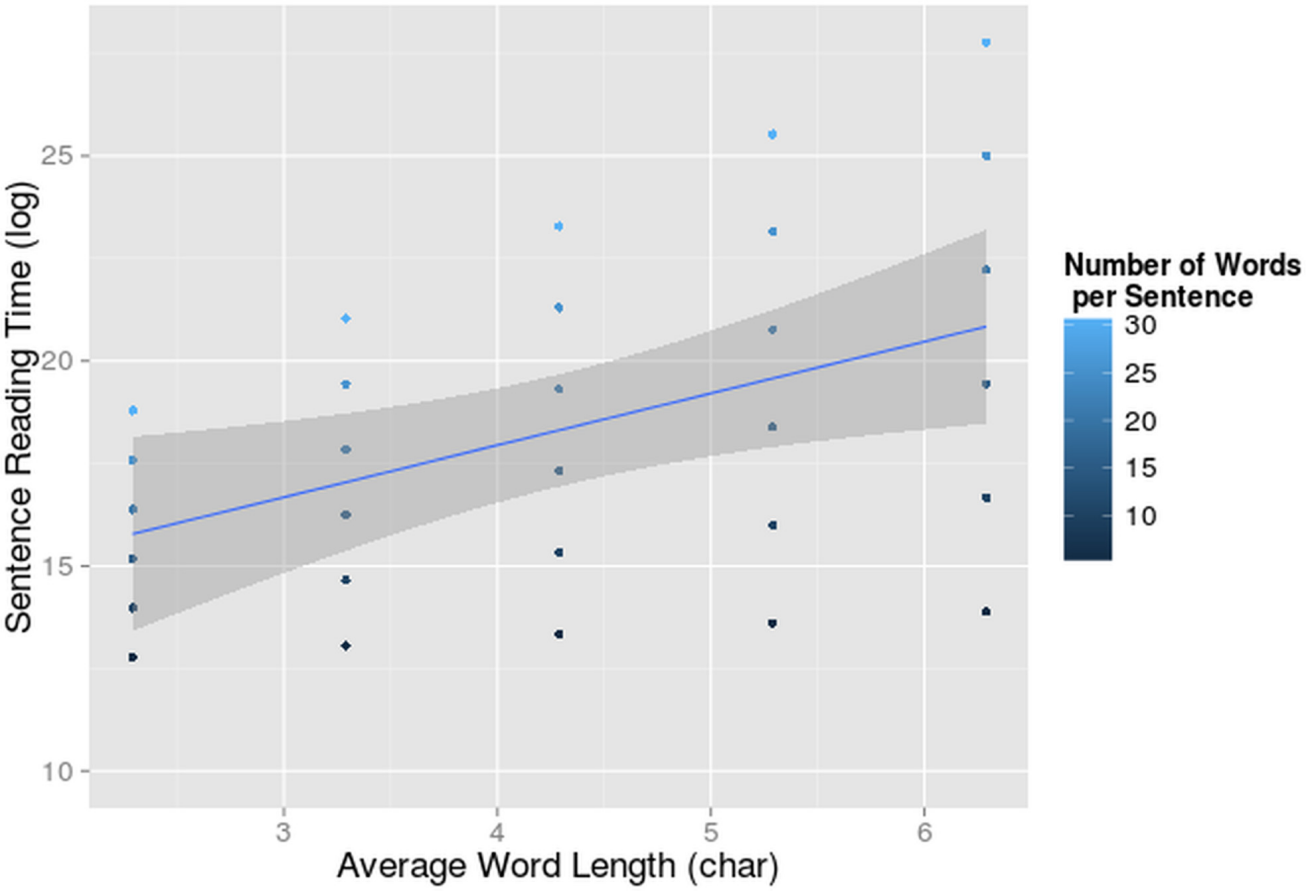
Interaction-effect of number of words and word length on reading times. Sentence reading time (log-transformed on the y-axis) in function of average word length per sentence (on the x-axis) and number of words per sentence. The 95% confidence interval for the main effect of word length is indicated in grey.

A main effect of word length (F = 19, df = 232.71, p<0.001) and number of words per sentence (F = 80.89, df = 21.84, p<0.001) was found. Obviously, longer reading times were found with sentences with longer words and more words. The interaction between these two variables was also significant (F = 14.20, df = 233.24, p<0.001). They reinforce each other’s effect (Fig 2). Apparently long sentences add an additional cost to the reading process when reading long words and do so more for L2 than L1. We did not find a main effect of L2 proficiency on sentence reading time or an interaction of L2 proficiency with language. In our dataset there was no evidence that L2 reading speed was altered by L2 proficiency.

None of the 3-way, 4-way or 5-way interactions contributed significantly to the fit of the model (all χ^2^ < 2.01).

#### Number of Fixations per Sentence

Sentences with fixation counts more than 3 standard deviations from the subject means were excluded (2.15%). The fixation counts per sentence were analyzed with a generalized linear mixed model with a Poisson distribution.

**Fig 3:**
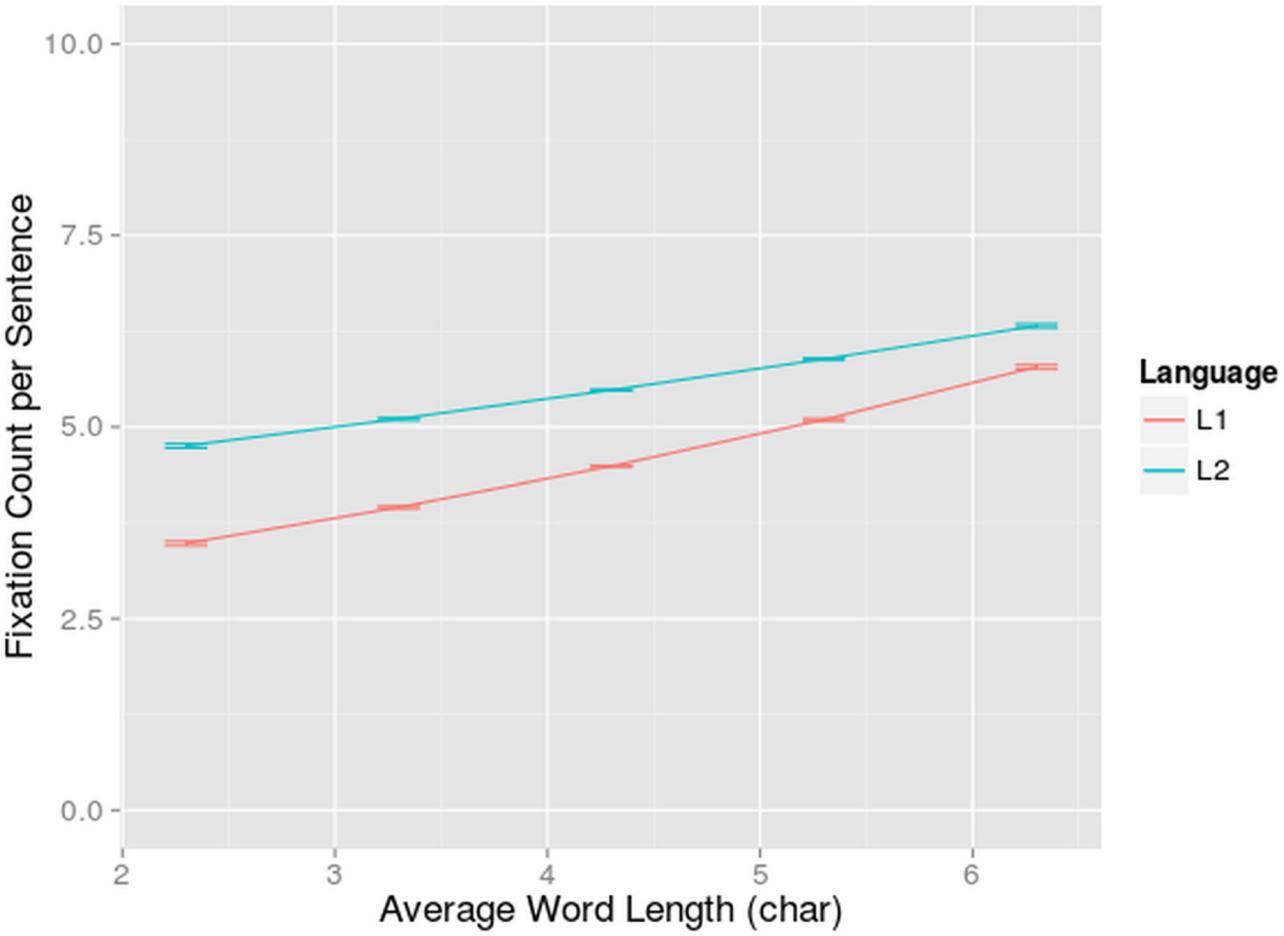
Interaction-effect of language and word length on fixation count. Fixation count per sentence (on the y-axis) in function of average word length per sentence (on the x-axis) for bilinguals reading in L1 and L2. The standard errors are indicated by whiskers on the graph.

A main effect of language was found (β = 0.200, z = 6.87, p<0.001): bilinguals made 13% more fixations in their L2 than in their L1 (6.75 fixations compared to 5.88 fixations). The E-Z reader model predicts more fixations when words get longer. Indeed, a main effect of word length (β = 0.168; z = 3.92, p<0.001) was found. A main effect of number of words (β = 0.101; z = 28.73, p<0.001) was also found, which interacted significantly with word length (β = 0.0170; z = 3.03, p<0.005). Again in longer sentences, the burden put on the reader by longer words increased for reading in L1 and L2. The word length effect was present both in L1 and L2 reading, but behaved in a different way: a significant interaction was found between language and word length (β = -.0555; z = -2.43, p<0.05). The effect of word length was smaller for L2 reading and the difference in fixation count for L1 versus L2 was smaller in the sentences with the longer words. This might be explained by the slower lexical processing in L2. When reading in L2, the eyes stayed on a certain word, short or long, for a longer period of time. This might have limited the need for a second fixation to longer words in L2, relative to L1 (Fig 3).

**Fig 4:**
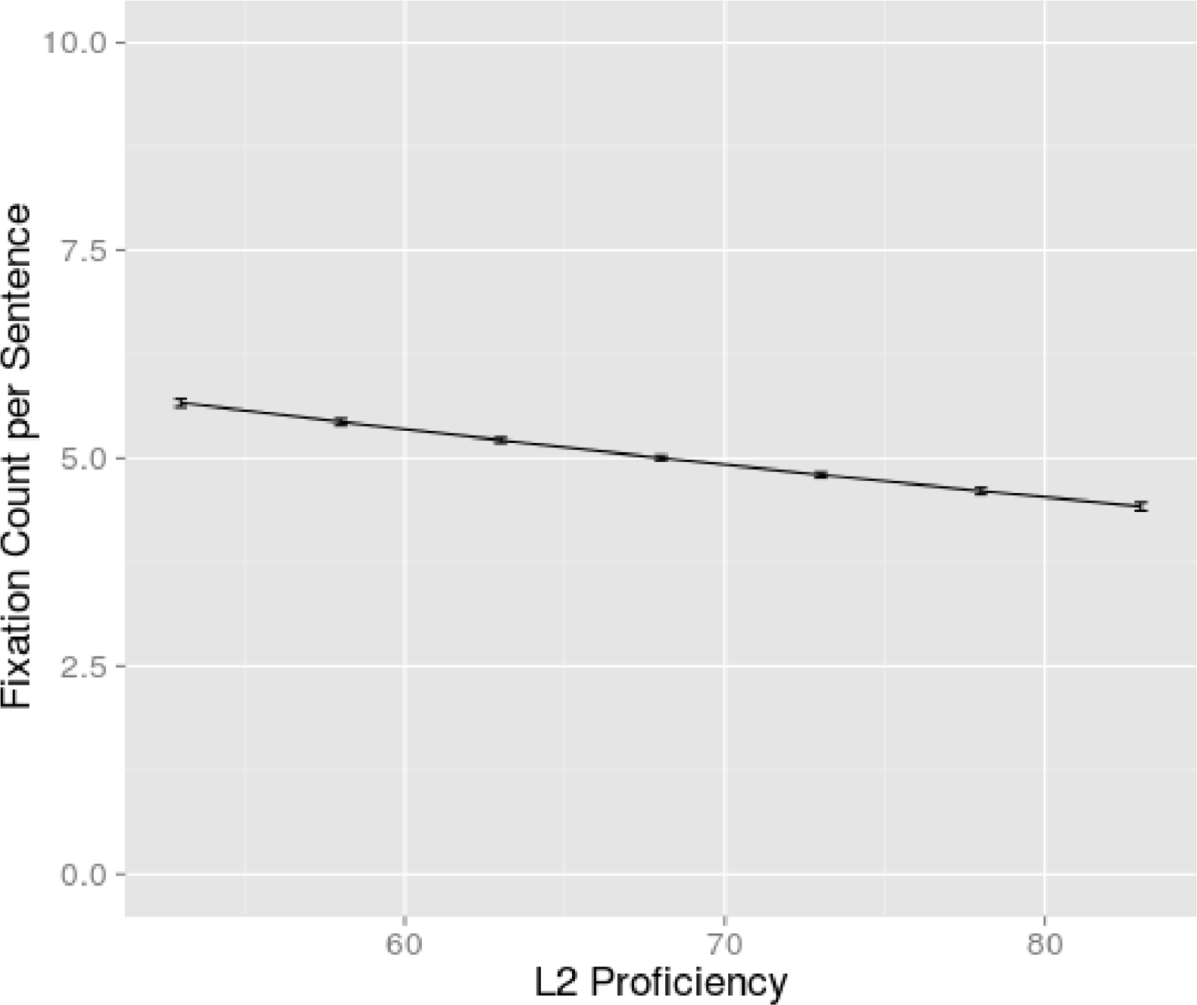
Effect of L2 Proficiency on Fixation Count. Fixation count per sentence (on the y-axis) dependent on the participant’s L2 composite proficiency score (on the x-axis). The 95% confidence interval is indicated by the dotted lines.

A main effect of L2 proficiency (β = -0.00828; z = -2.21, p<0.05) was also found (Fig 4). As L2 proficiency increased, the number of fixations decreased, also when reading in the mother tongue. This is not surprising because the correlation between the proficiency in L1 and in L2 was 0.76. It is important to note that the interaction between language and proficiency was not significant: even for the bilinguals who are very proficient in their L2, the fixation count was higher in L2 than in L1. The participants scoring 50%-65% on their L2 proficiency fixated on average 6.73 times. The participants scoring above 70%-85% fixated on average 5.79 times. None of the 3-way, 4-way or 5-way interactions contributed significantly to the fit of the model (all χ^2^<3.24).

#### Average Fixation Duration

Sentences with an average fixation duration differing more than 3 standard deviations from the general mean were excluded (8.64%).

A main effect of language was found (F = 22.06, df = 193.61, p<0.001): bilinguals fixated on average 9% or 20ms longer in their L2 than their L1 (238.72ms compared to 218.74ms). This explains the effect that we found when analyzing the Sentence Reading Times: longer sentences prolonged the reading time significantly more in L2 than in L1. For each fixation, extra time was added to the total sentence reading time. Because this additional time was longer for L2, we got a steeper incline in reading time. This finding combined with the higher fixation count in L2 is compatible with a child like reading pattern in L2, caused by a slower second language processing.

A main effect of number of words (F = 7.3, df = 62.4, p<0.01) was found and this variable interacted with language (F = 14.57, df = 195.87, p<0.001). This interaction shows us that only in L2, the average fixation durations were longer when the sentences were longer.

**Fig 5:**
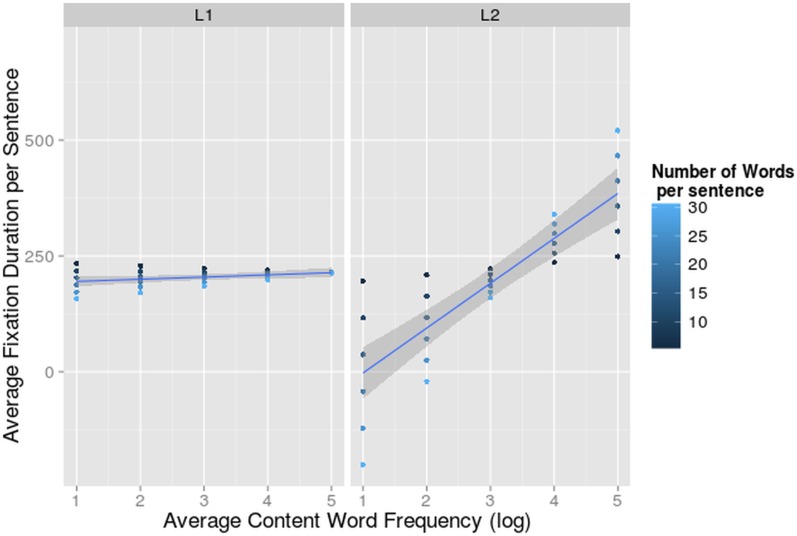
Interaction-effect of Word Frequency and Number of Words on Fixation Duration. Average fixation duration per sentence (on the y-axis) dependent on average content word frequency per sentence (log-transformed on the x-axis) and number of words per sentence for Bilinguals reading in L1 and L2. The 95% confidence interval of the main effect of content word frequency per language is indicated in grey.

The 3-way interaction between language, number of words and frequency (F = 6.41, df = 201.91, p<0.05) was significant (Fig 5).

Word frequency is the most frequently investigated determinant of word fixation times. Low frequency words normally yield longer fixation durations, but because we were looking at the average fixation duration including re-fixations and skips, we expected a reversed effect. A high frequent word might receive just a single fixation, while more difficult, less frequent words might receive two or even three fixations. These fixations will be shorter than the single one, but the sum of the two will be longer [91]. Indeed, in L2 we found this reversed frequency effect in sentences that contain more than 9 words. When the average content word frequency was low, i.e. sentences with more difficult words, bilinguals fixated shorter on average.

We did not detect this frequency effect in L1, probably because most words received just a single fixation (74.76% of the fixated words in L1 versus only 65.82% of the fixated words in L2).

**Fig 6:**
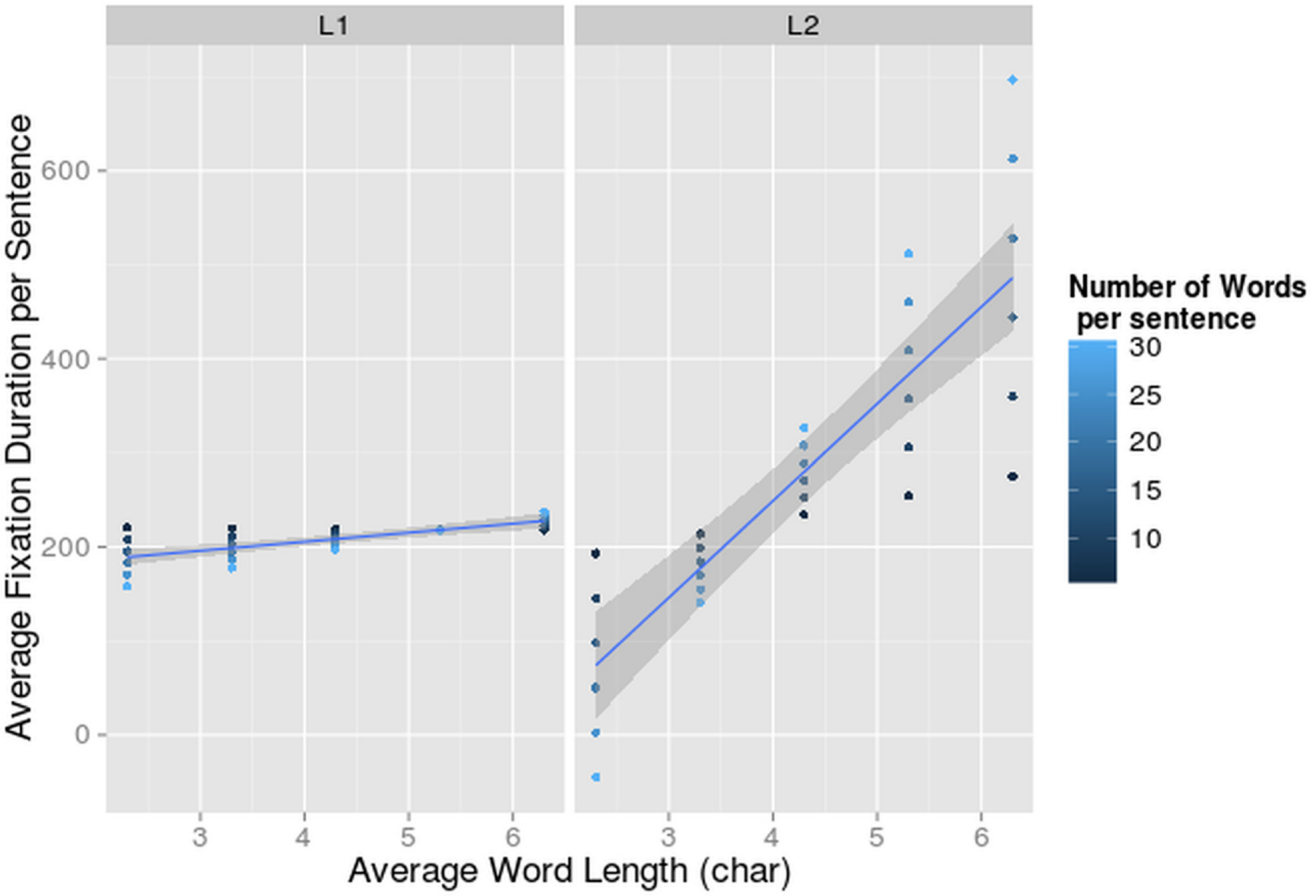
Interaction-effect of Number of Words, Word Length and Language on Fixation Duration. Average fixation duration per sentence (on the y-axis) dependent on average word length per sentence (on the x-axis) and number of words per sentence for bilinguals reading in L1 and L2. The 95% confidence interval for the main effect of word length per language is indicated in grey.

The interaction between language and word length also reached significance and indicated that there was an effect of word length (F = 8.18, df = 195.87, p<0.01) only when reading in L2, and more specifically that in sentences with longer words the average fixation duration was longer (Fig 6).

The 3-way interaction between language, number of words and word length (F = 6.62, df = 195.84, p<0.05) was significant (Fig 6). In L2, the effect of word length was bigger in sentences with more words. This resulted in inflated fixation durations when long words were positioned in long sentences. In sentences containing very short words, fixation durations were longer in short sentences. In sentences with short words the fixations get shorter in longer sentences, and in sentences with long words the reverse happens. This means that longer words, pose a larger burden on the reading and language processing mechanisms when reading in L2 than in L1. Again L2 proficiency did not influence the average fixation duration of our participants, while reading in L1 or L2. None of the 4-way or 5-way interactions contributed significantly to the fit of the model (all χ^2^ < 2.65).

#### Rightward Saccade Length

We analyzed the average saccade length per sentence of the saccades that were directed to the right. The saccades during which the participant blinked and sentences with an average saccade length differing more than 3 standard deviations from the general mean were excluded (1.67%). The Box-Cox method [90] determined that the log transformation of the variable was optimal to achieve a normal distribution. This log of the average saccade length was analyzed.

A main effect of language was found (F = 30.77, df = 66.56, p<0.001): bilinguals moved their eyes across 12% shorter distances when reading in L2 than in L1 (8.30 compared to 9.35 characters). This result is again in line with our child like reading hypothesis and ties in with the fact that more fixations were made in L2. It has been shown that reading skill influences the size of the perceptual span seeing that beginning readers have smaller perceptual spans than more skilled readers [5, 75]. It is plausible to assume that the same is going on for participants reading in their L2. Because of this smaller perceptual span, less parafoveal processing is possible and people move their eyes more close to their previous fixation. The risky reading strategy that we hypothesized, states that bilinguals might make longer saccades and skip more words in L2. Our bilingual participants did not seem to do that.

A main effect of number of words (F = 17.35, df = 98.84, p<0.001) was found. Participants moved their eyes further in sentences with more words. Balota, Pollatsek & Rayner showed that readers skipped more words when they were predictable in the sentence context [92]. This causes participants to make longer saccades. It is probable that words are more predictable in long sentences because the preceding sentence context is more semantically restrictive, but this requires further investigation.

Where to move the eyes is strongly influenced by low-level variables like word length and space information. Longer words usually lead to longer saccades [93]. We did not find an effect of word length. This is due to the fact that we include both intra-word and inter-word saccades in this analysis. This means that for long words, that were often fixated more than once, saccades were shorter. This probably balances out the effect that we would find for the inter-word saccade length, namely that long words would elicit longer saccades.

**Fig 7:**
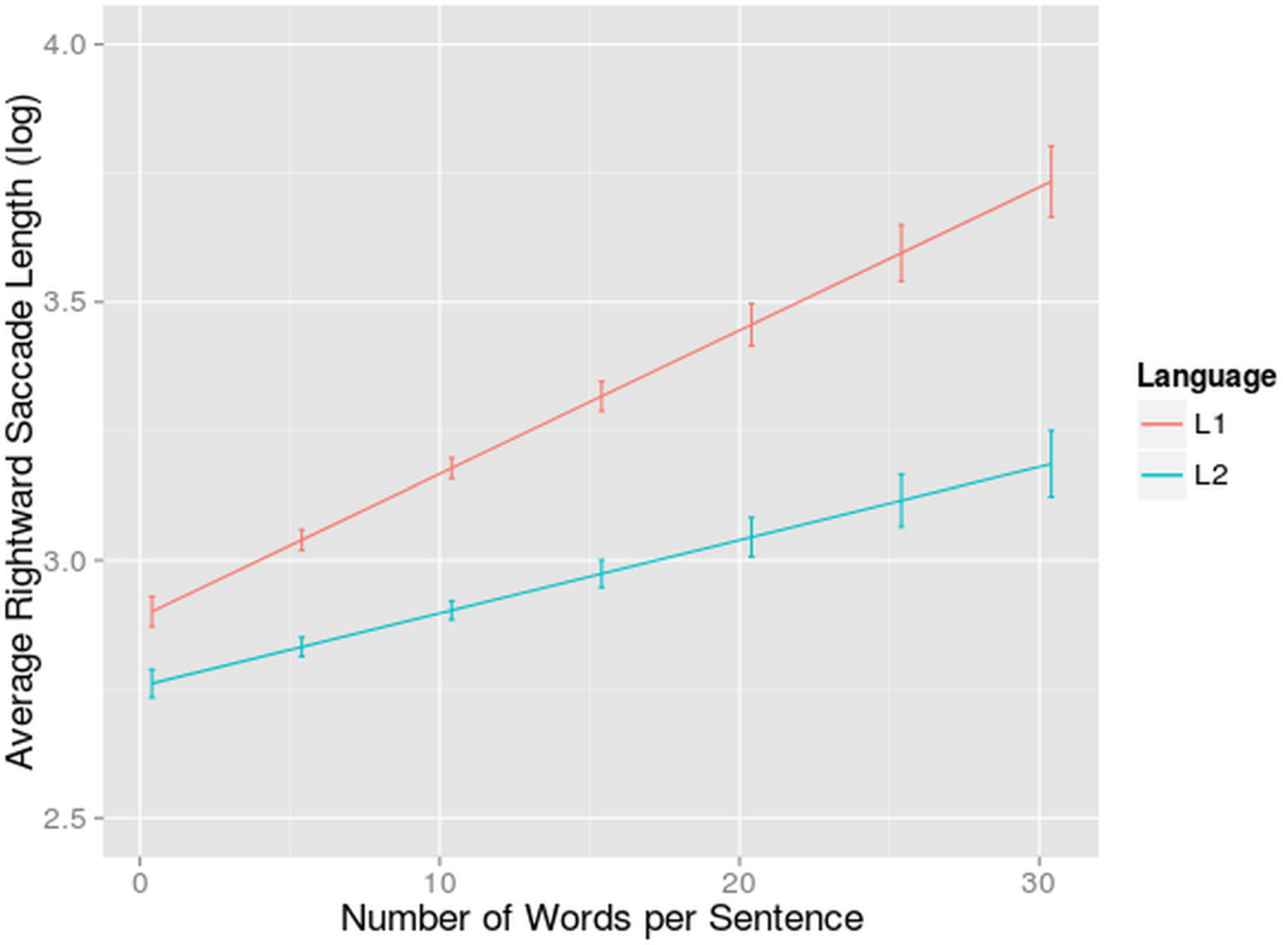
Interaction-effect of Language and Number of Words on Saccade Length. Average saccade length per sentence (on the y-axis) dependent on average number of words per sentence (on the x-axis) for bilinguals reading in L1 and L2. Standard errors are indicated with whiskers on the graph.

A significant interaction was found between language and number of words (F = 4.60, df = 151.58, p<0.05). This suggests a differential number of words effect. In other words, the difference between saccade length in L2 and L1 reading was bigger for sentences with more words (Fig 7). This could point towards the fact that when reading in L2, participants predicted less of the upcoming words than when reading in L1. None of the 3-way, 4-way or 5-way interactions contributed significantly to the fit of the model (all χ^2^ < 2.57).

#### Skipping Rate

The probability of skipping a word in the first pass was analyzed. We fitted a linear mixed effect model with a binomial distribution.

The main effect of language was significant (β = -0.202; z = -4.180, p<0.001). In line with expectations, participants skip 5% more words when reading in their L1 (52.22%) compared to reading in their L2 (47.62%). Skipped words are thought to be processed on the fixation prior to the skip, when the word was still in the parafovea, and in part after the skip [17, 22]. We found that bilinguals skip fewer words when they read in their least proficient language. This result was thus in line with slower language processing in L2, allowing less time for the parafoveal processing of the next word when reading, resulting in less skipping. This does not point towards the possibility that bilinguals might use a risky reading strategy when reading in L2 [30].

Word length has been found to be the most important determinant of word skipping [34]. Very short words were skipped fairly often, while words of 9 or more characters were almost never skipped. We indeed found an effect of word length on skipping rate (β = -0.120; z = -4.104, p<0.001). More specifically: When sentences contained longer words, the probability of skipping those words was lower. None of the interactions contributed significantly to the fit of the model (all χ^2^ < 1.73).

#### Regressions Rate

Finally, probabilities of making a regressive eye movement were analyzed. The saccades during which the participant blinked were excluded from the analyses. A saccade was considered a regression when the eye moved from a word further in the sentence to a previous word (intra word regressions were not entered in the analyses). We fitted a linear mixed effect model with a binomial distribution.

**Fig 8:**
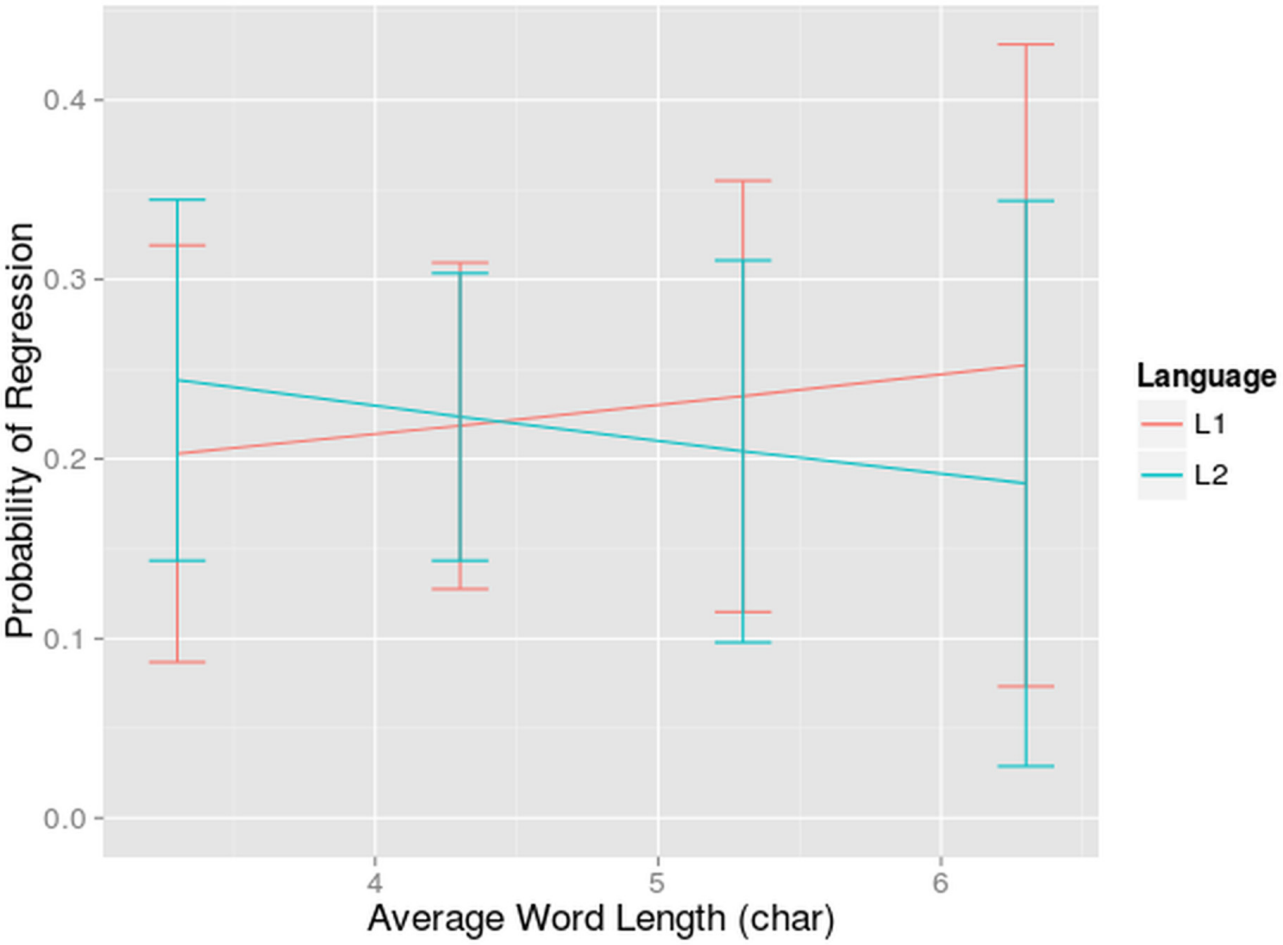
Interaction-effect of Language and Word Length on Regression Rate. The probability of making a regression (on the y-axis) dependent on the average word length per sentence (on the x-axis) for reading in L1 and L2. The standard errors are indicated by whiskers on the graph.

The E-Z reader model states that regressions occur when there is difficulty with integrating a certain word in the current sentence context. This means that comprehension difficulties while reading a text can change the eye movement behavior. For example, when participants read garden-path sentences, they make more regressions to earlier parts of the text [94]. Although we expected that L2 readers would make more regressions, we did not found a higher regression rate when bilinguals read in their L2. No main effect of language was found (bilinguals made a regressive saccade in 22.63% of the cases in L1 and 24.07% of the cases in L2). The only significant effect was the interaction between language and word length (β = -0.208, z = -2.039, p<0.05). In our data L2 readers do regress more than L1 readers, as expected, but only in sentences that contain relatively short words (on average 3.3 characters or less). In the more complex, longer sentences bilinguals made the same amount of regressions when reading in their L1 as in L2. When reading in L1, the longer the words, the more regressions were made (Fig 8). This could be expected, because these words are usually harder to process, and more integration difficulties are likely to arise. This relationship reversed in L2. This pattern of more regressions towards short words can be explained by the fact that short words were skipped more often. It is thus more likely that such a word was not processed sufficiently and therefore that the reader has to return to that word. Although both patterns are plausible, it is still an open question why we found the former when bilinguals read in L1 and the latter when bilinguals read in L2. This might be because the average fixation duration was longer in L2 than in L1, especially in sentences with longer words. This means that the chance that a long word was not sufficiently processed in a first pass reading was lower in L2 than in L1. None of the 3-way, 4-way or 5-way interactions contributed significantly to the fit of the model (all χ^2^ < 2.31).

For a full summary of the averages and standard deviations of the eye movement variables for L1 and L2 reading, see Table 3.

**Table 3:** Eye movement variable averages for young and older children and adults from Rayner’s [5] and Blythe et al.’s [4] study and eye movement variable averages for bilingual L1/ L2 and monolingual reading. Differences between the means are reported in the last two columns [percentage] in each section.

	Rayner [5]						Blythe et al. [4]					Our data				
Variable	7–8 year olds	11–12 year olds		adults	young children—adults	older children-adults	7–9 year	10–11 year	adults	young children-adults	older children-adults	monolingual	bilingual L1	bilingual L2	mono-L1	L2—L1
Sentence Reading Time (ms)	-	-	-		-	-	5473	4666	2965	2508 (84.6%)	1701 (57.4%)	1279.34 [1030.49]	1254.41 [1073.06]	1522.98 [1293.51]	24.93 (1.9%)	268.6 (17.6%) ***
Fixation Count per sentence	15	8	6		9 (150%)	2 (33.3%)	16.8	15.6	10.3	6.5 (63.1%)	5.3 (51.5%)	5.63 [4.59]	5.59 [4.83]	6.75 [5.77]	0.04 (0.7%)	1.16 (17.2%) ***
Average Fixation Duration (ms)	280	240	235		45 (19.1%)	5 (2.1%)	285	256	249	36 (14.5%)	7 (2.8%)	217.28 [44.74]	213.42 [42.47]	238.72 [109.74]	3.86 (1.8%)	25.3 (10.6%) ***
Saccade length (characters)	2.8	6.4	6.8		-4 (-58.8%)	-0.4 (-5.9%)	-	-	-	-	-	10.09 [3.58]	9.45 [3.24]	8.30 [2.54]	0.64 (6.3%)	-1.15 (-13.9%) ***
Average skipping probability (%)	-	-	-		-	-	39	44	44	-5 (-11.4%)	0 (0%)	51.99 [49.96]	52.27 [49.95]	47.62 [49.95]	-0.28 (-0.5%)	-4.65 (-9.8%) ***
Average regression probability (%)	-	-	-		-	-	-	-	-	-	-	25.23 [43.43]	22.58 [41.81]	24.07 [42.75]	2.65 (10.5%)	1.49 (6.1%)

* p<0.05

** p<0.01

*** p<0.001

### Bilingual L1 reading vs. Monolingual reading

#### Sentence Reading Time

Sentence reading times that differed more than 3 standard deviations from the general mean reveal unusual distraction and were therefore excluded from the analysis (4.06%). Sentence reading times were log transformed as suggested by the Box-Cox method [90] to obtain a normal distribution and then analyzed with the linear mixed model described above.

We did not find a main effect of bilingualism (F = 2.46, df = 49.9, p = 0.123). Monolinguals read sentences in 1.28s, bilinguals in 1.25s. In order to exclude the possibility that this null effect was due to the use of a restricted (optimally matched on average word length, average word frequency and number of words per sentence) sentence set (n = 210), we also analyzed sentence reading times of the translation equivalent sentence set (n = 4 804). None of the interactions with the factor of bilingualism reached significance. The main effect of bilingualism was also not significant (F = 1.55, df = 49, p = 0.22). This means that, in this dataset of natural reading, there is no evidence for a slower reading process on a sentence-level for bilinguals in L1 compared to monolinguals in L1. This finding is of great relevance, given that some recent studies in word production and word recognition suggested a considerable speed disadvantage for bilinguals. Gollan et al. [65] and Ivanova and Costa [68] found about 33-60ms (5–10%) slower L1 picture naming for bilinguals compared to monolinguals. In the visual word recognition domain, Lehtonen et al. [72] and Randsell and Fischler [71] found 80 to 170ms (13–25%) slower L1 lexical decision times for bilinguals compared to monolinguals. This would correspond to a large difference of 166-320ms in sentence reading times here, which we did not find for natural reading.

**Fig 9:**
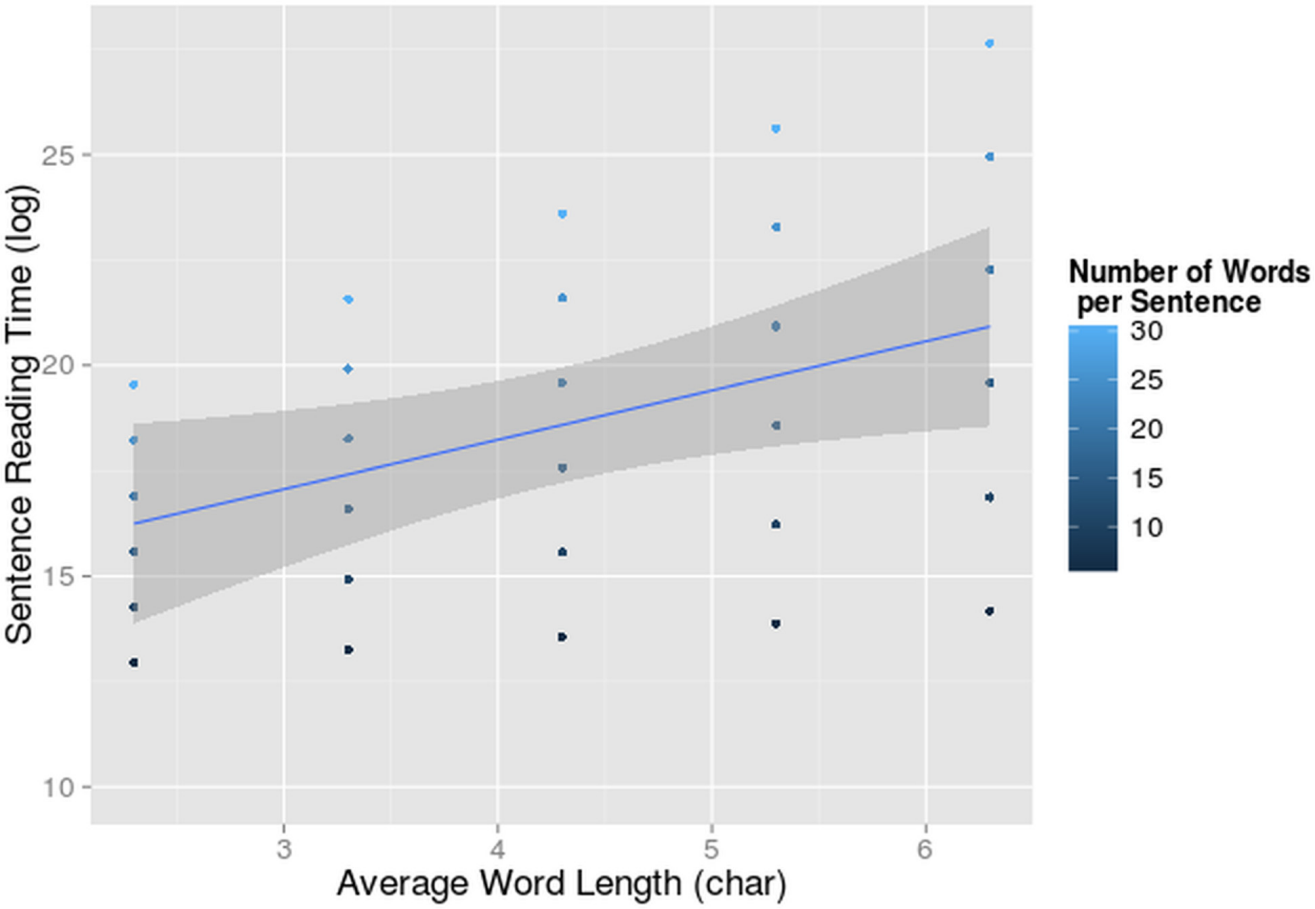
Interaction-effect of Word Length and Number of Words on Reading Time. The sentence reading time (log-transformed on the y-axis) dependent on average word length per sentence (on the x-axis) and number of words per sentence for monolinguals and bilinguals reading in L1. The 95% confidence interval for the main effect of word length is indicated in grey.

We found a main effect of number of words (F = 852.29, df = 166.76, p<0.001), of word length (F = 17.45, df = 264.1, p<0.001) and a significant interaction between the two (F = 12.86, df = 253.07, p<0.001). Again these two variables reinforced each other’s effect, so that in longer sentences the length of the words had a larger effect on sentence reading time (Fig 9).

**Fig 10:**
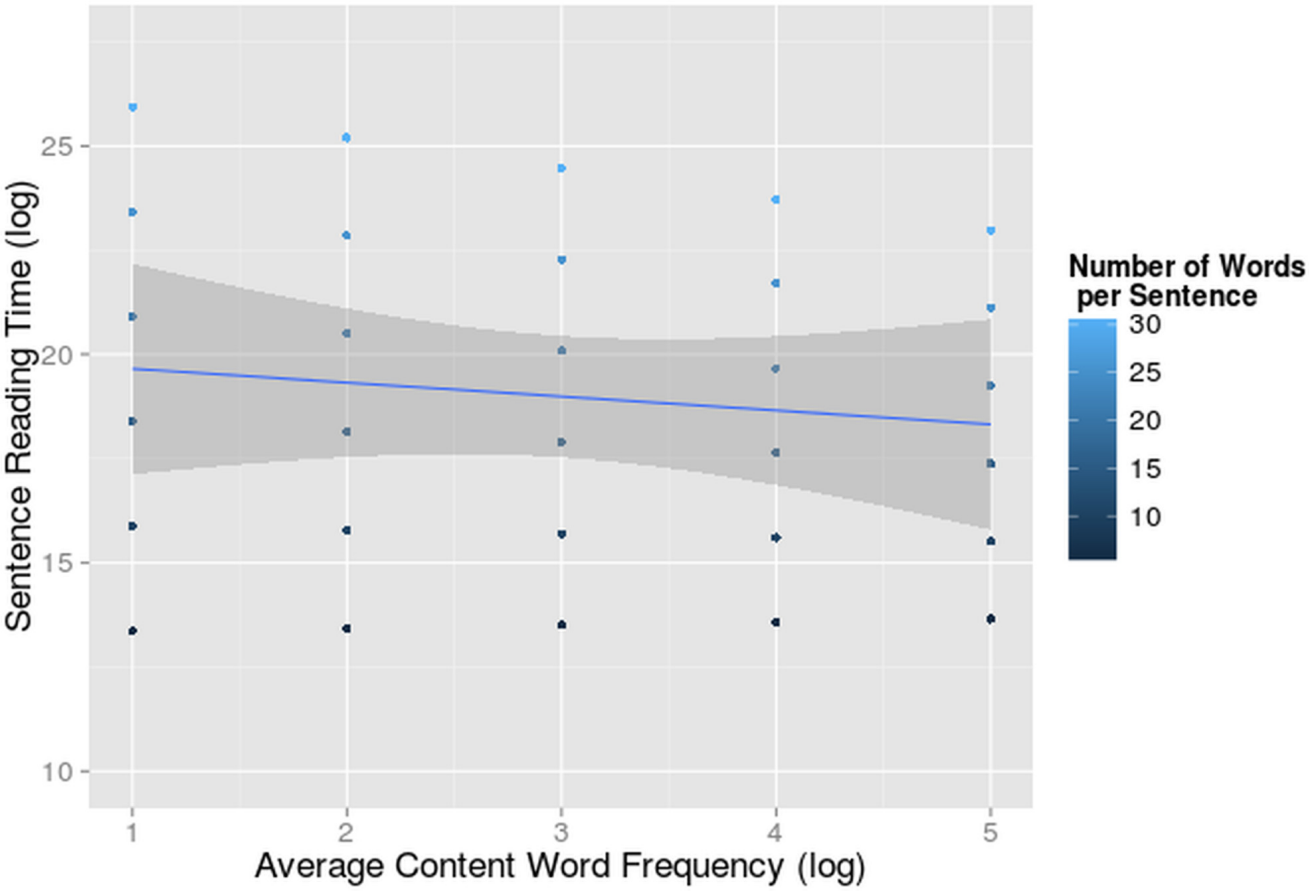
Interaction-effect of Word Frequency and Number of Words on Reading Time. Sentence reading time (log transformed on the y-axis) in function of average content word frequency per sentence (log transformed on the x-axis) and number of words per sentence for monolinguals and bilinguals reading in L1. The 95% confidence interval of the main effect of content word frequency is indicated in grey.

We found a significant interaction between number of words and frequency (F = 4.05, df = 197.36, p = 0.045) indicating that participants read faster when the content words of a sentence were more frequent, but only in longer sentences (Fig 10). Reading time is a cumulative variable, so the difference between high and low frequency sentences probably only reached significance when there were enough words to be processed. In fact, the (sentence-level) frequency effect was even absent in sentences shorter than 9 words. We have to consider that the frequency measure we used in these models is a very coarse one. Given our focus on sentence-level effects, frequency is averaged over content words, but we do look at the reading time of all the words in the sentence. So this makes the frequency effect hard to detect. Indeed, in a recent paper we showed strong word-level frequency effects for bilinguals and monolinguals in the same eye-tracking corpus [79]. None of the 3-way, 4-way or 5-way interactions contributed significantly to the fit of the model (all χ^2^ < 1.37).

#### Number of Fixations per Sentence

Sentences with fixation counts differing more than 3 standard deviations from the subject means were excluded (2.15% for the L1-L2 comparison and 0.4% for the L1-monolingual comparison). The fixation counts per sentence were analyzed with a generalized linear mixed model with a Poisson distribution.

The main effect of bilingualism was not significant. Monolinguals fixated on average 5.63 times, while bilinguals reading in L1 fixated on average 5.59 times, almost exactly the same. Native language reading yielded the same amount of fixations for bilinguals and monolinguals. A main effect of number of words (β = 0.106, z = 26.26, p<0.001) and word length (β = 0.151, z = 3.79, p<0.001) was found. Sentences that contain more words or longer words, received more fixations. The interaction between these two variables was also significant (β = 0.0103, z = 2.00, p<0.05): They strengthened each other’s effect. Although the effect of the number of words in a sentence was present for all word lengths, we only found a word length effect in sentences with more than 9 words.

**Fig 11:**
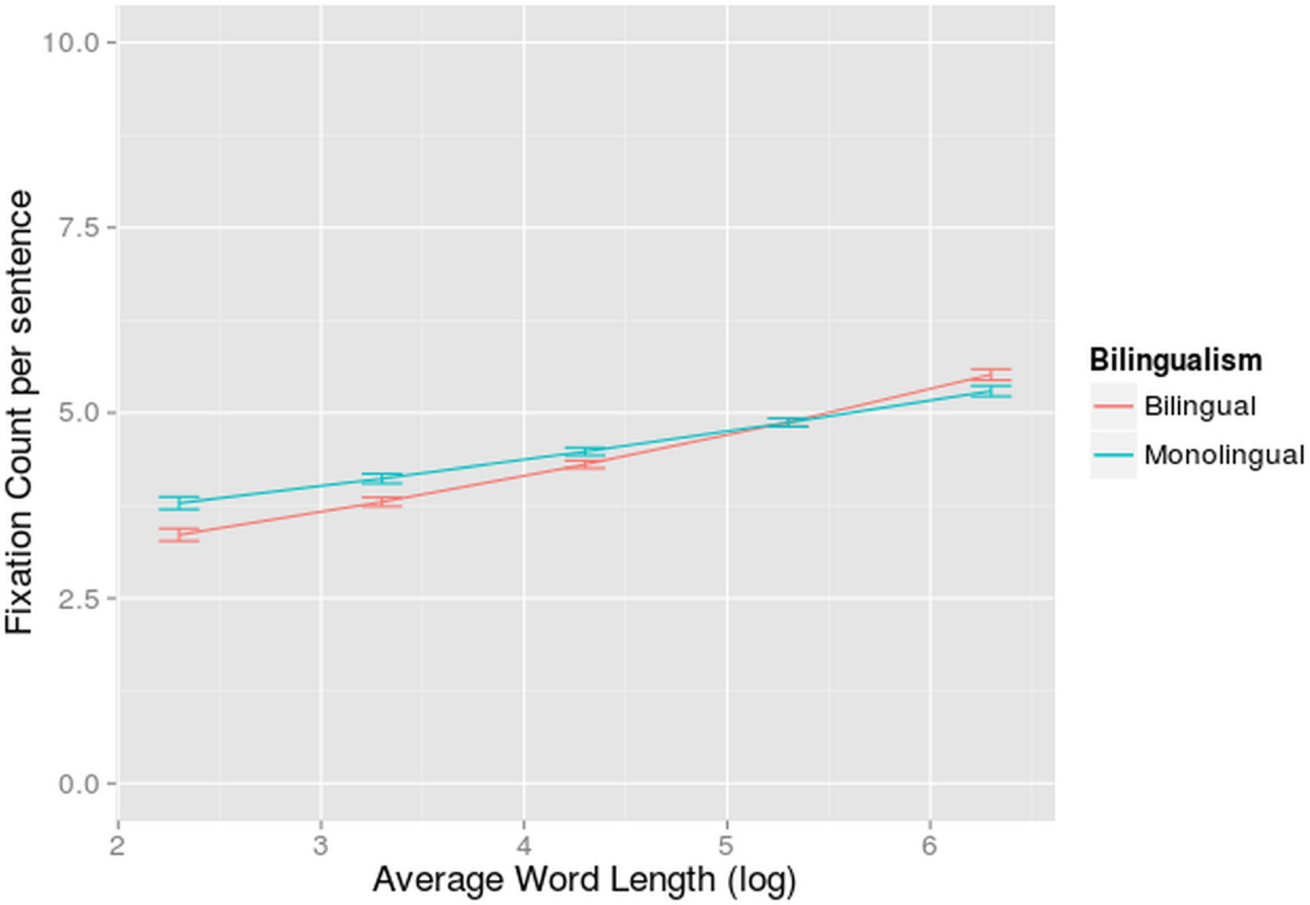
Interaction-effect of Word Length and Bilingualism on Fixation Count. Fixation count per sentence (on the y-axis) in function of average word length per sentence (on the x-axis) for bilinguals reading in L1 and monolinguals (separate regression lines). Standard errors are indicated by whiskers on the graph.

**Fig 12:**
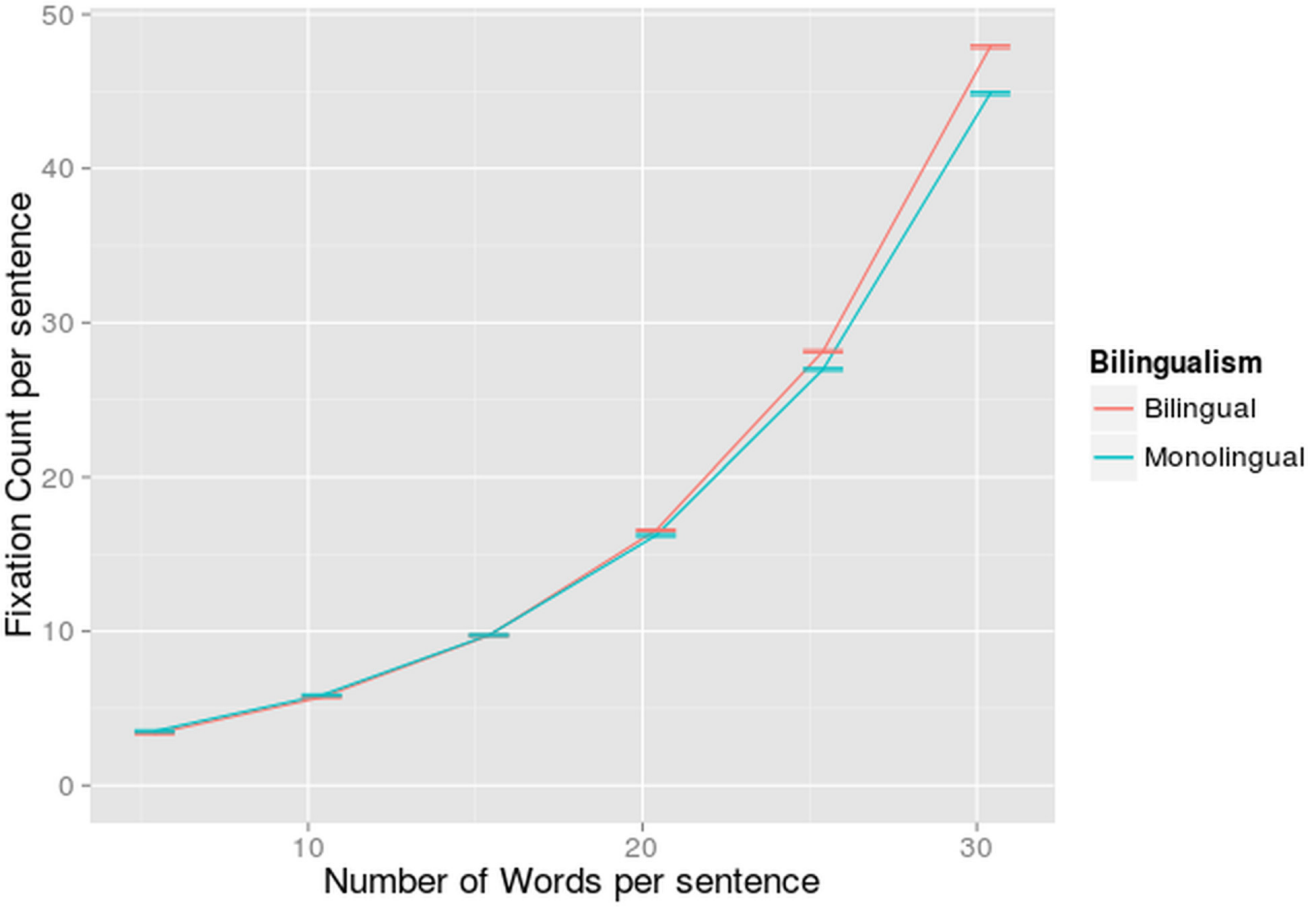
Interaction-effect of Number of Words and Bilingualism on Fixation Count. Fixation count per sentence (on the y-axis) in function of number of words per sentence (on the x-axis) for monolinguals and bilinguals reading in L1 (separate regression lines). The standard errors are indicated by whiskers on the graph.

A significant interaction between bilingualism and word length was also found (β = 0.0403, z = -2.00, p<0.05). Bilingualism also interacted significantly with number of words per sentence (β = -0.00451, z = -2.46, p<0.05). In both cases the effects of the latter variable was larger for the bilinguals compared to the monolinguals, although both were reading in their first language (Figs 11 and 12). The average word length of the sentences had a larger impact on how many times a participant fixates in a certain sentence when this participant is a bilingual than when he is a monolingual. Sentences with an average word length smaller than 5 characters were fixated less and sentences with an average word length larger than 5 were fixated more by bilinguals than by monolinguals. Also, bilinguals needed to fixate slightly more in long (more than 20 words) sentences compared to monolinguals, but this effect was relatively small.

**Fig 13:**
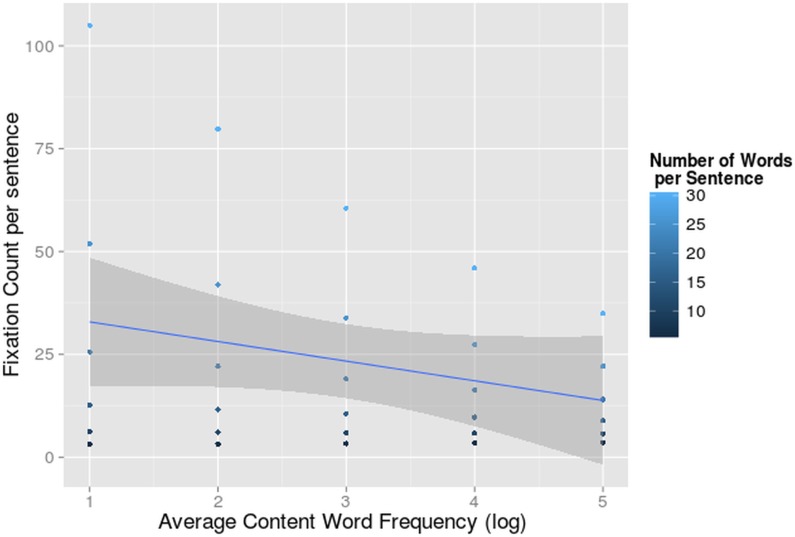
Interaction-effect of Word Frequency and Number of Words on Fixation Count. Fixation count per sentence (on the y-axis) in function of average content word frequency per sentence (log-transformed on the x-axis) and average number of words per sentence. The 95% confidence interval of the main effect of content word frequency is indicated in grey.

We also found a significant interaction between frequency and number of words (β = -0.0125, z = -2.59, p<0.01): In long sentences we found a frequency effect. This means that there were more fixations in the sentences with a lower average word frequency (Fig 13). In short sentences this effect was absent. Because of the focus on sentence-level effects, the average content word frequency measure we used is not a sensitive measure and would be even less accurate for shorter sentences. The effect was enlarged because fixation count is a cumulative variable. A more sensitive word level analysis will probably reveal larger and more ubiquitous frequency effects. None of the 3-way, 4-way or 5-way interactions contributed significantly to the fit of the model (All χ^2^ <1.3).

#### Average Fixation Duration

Sentences with an average fixation duration differing more than 3 standard deviations from the general mean were excluded (6.06%).

No effect of bilingualism was found. Bilinguals fixated on average 213.42ms in their L1 and monolinguals fixated on average for 217.28ms. Being a bilingual did not alter the durations of the fixations. None of the effects contributed significantly to the fit of the model (All χ^2^ <2.73).

#### Rightward Saccade Length

We analyzed the average saccade length per sentence of the saccades that were directed to the right. The saccades during which the participant blinked were excluded from the analyses. Sentences with an average saccade length more than 3 standard deviations from the general mean were excluded (4.69%). The Box-Cox method [90] determined that the log transformation of the variable was optimal to achieve a normal distribution. This log of the average saccade length was analyzed.

The effect of bilingualism was not significant. Bilinguals reading in L1 did not move their eyes further than monolinguals (9.45 characters for bilinguals and 10.09 characters for monolinguals).

There was a significant effect of number of words (F = 53.12, df = 90.09, p<0.001). In longer sentences, longer saccades were made. Again this might have been due to the end of sentences being more predictable than the beginning, making saccades longer the further you progress in that sentence.

The effect of L1 proficiency was marginally significant (F = 3.70,df = 25.18, p = 0.066). More proficient participants moved their eyes further. This finding makes clear, that the knowledge of another language does not change the saccade strategy of the reader. It might however be influenced by the knowledge and proficiency of the language you are reading in. Again, this can be related to the development of children where they develop larger saccades as they augment their language skill. None of the interactions contributed significantly to the fit of the model (All χ^2^ <3.45).

#### Skipping Rate

The probability of skipping a word in the first pass was analyzed. We fitted a linear mixed effect model with a binomial distribution.

**Fig 14:**
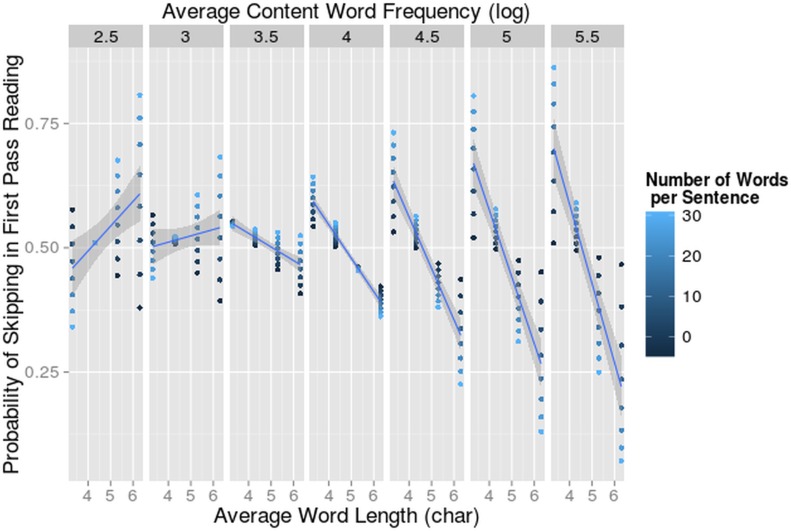
Interaction-effect of Word Frequency and Number of Words on Skipping Rate. The probability of skipping a word in first pass reading (on the y-axis) dependent on average word length per sentence (on the x-axis), number of words per sentence and average content word frequency per sentence (log-transformed in the separate panels). The 95% confidence intervals for the effects of word length per content word frequency value are indicated in grey.

We did not find a difference between the skipping probability for monolinguals (51.99%) and bilinguals reading in L1 (52.27%). Again as expected, the word length effect was significant (β = 0.202; z = -4.303, p<0.001). In sentences with longer words, the skipping rate was lower. There was also a significant effect of number of words (β = 0.00586; z = 2.792, p<0.01). In long sentences, the probability of skipping was higher than in short sentences. The 3-way interaction between number of words, word length and frequency was significant (β = -0.0222; z = -3.258, p<0.005). Sentences with longer words had a lower skipping rate, but this effect reversed in difficult, long sentences (two left panels of Fig 14). It seems that words were glossed over more when a sentence in L1, on a whole, became too difficult. When a sentence contained a lot of difficult words, the probability of skipping in those sentences with longer words was higher. None of 4-way or 5-way interactions contributed significantly to the fit of the model (All χ^2^ <1.74).

#### Regressions Rate

Finally, probabilities of making a regressive eye movement were analyzed. The saccades during which the participant blinked were excluded from the analyses. A saccade was considered a regression when the eye moved from a word further in the sentence to a previous word (intra word movements were not considered regressions). We fitted a linear mixed effect model with a binomial distribution.

No main effect of bilingualism was found (Regression rates 22.58% for bilingual L1 reading and 25.23% for monolingual reading). No other factors yielded significant effects. None of the interactions contributed significantly to the fit of the model (all χ^2^ <2.89).

For a full summary of the averages of the eye movement measures of L1 and monolingual reading see Table 3.

## General Discussion

We gathered a large comprehensive natural reading corpus of monolingual and bilingual eye movements. The goal of this study was twofold: a) to compare the eye movement pattern of bilinguals reading in L1 vs. reading in L2 and b) to compare the eye movement pattern of bilinguals reading in L1 vs. monolinguals reading in the mother tongue.

### Bilingual L1 vs. Bilingual L2

We found clear sentence-level differences between L1 and L2 reading. In line with our expectations, and in concordance with the hypothesis of more child-like reading, we observe: a) 17.6% longer sentence reading times, b) 17.2% more fixations per sentence, c) 10.6% longer fixation durations, d) 13.9% shorter saccade lengths and e) a 9.8% lower probability of skipping a word in L2 compared to L1 reading (for more details see Table 3). Hence, slower sentence reading times in L2 were due to a higher amount of fixations, which were longer and closer together, and to the fact that fewer words were skipped.

#### Comparison with eye movement pattern of children

We predicted that the eye movement pattern of bilinguals reading in L2 would resemble the eye movement pattern of another kind of language learners, namely children. We will compare our L1-L2 results with Rayner’s [5] and Blythe et al’s [4] results of eye tracking studies in which children read sentences (summary in Table 3). Rayner tested three groups of children (7–8, 9–10 and 11–12 year olds) and adults. Each group read text material taken from textbooks suited for second grade children. Blythe et al. showed 3 groups of participants (adults, 7–9 year old children and 10–11 year old children) the same set of constructed sentences. We must note that the sentences that Blythe et al. presented were between 70–80 characters long and the ones that Rayner presented were 25–37 characters long, while ours were on average 32 characters long (56% shorter than Blythe’s). The differences in the absolute size of fixation count and reading time between our data and Blythe et al.’s are probably due to this difference in sentence length. As you can see in Table 3, our L1 sentence reading times and fixation counts are about 55% lower than Blythe et al.’s adult sentence reading time and fixation count, while the adult fixation count in Rayner’s study was comparable to our L1 fixation count.

Looking at Table 3, it is clear that the changes that L2 reading causes in the eye movement behavior are similar to, and in the same direction as, the changes that reading as a child, or an L1 learner entails: Sentence reading times, average fixation duration and fixation count increase, while rightward saccade length decreases for children compared to adults and for L2 readers compared to L1 readers. The exception is that Blythe et al.[4] did not find a significant effect for skipping rates, while we did find less skipping for bilinguals reading in L2. A more recent study by Blythe et al. [73] and one by Haïkiö et al. [75] did find a decrease in skipping rate of about 55% for younger children and 20% for older children compared to adults. Another difference is that we did not find a difference between the regression rates for reading in L1 and reading in L2, while the largest part of the studies of children’s eye movement studies found a higher regression rate for children [4–6, 73, 74, 77, 78]. In our data, the regression rate was only slightly higher in L2 than in L1 and only when the participant reads sentences containing short words. It is known that regression rates indicate integration difficulty. It is possible that because our participants have a relatively high L2 proficiency, they did not have more integration difficulties when reading in L2 compared to reading in L1 (which was confirmed in the text comprehension scores), while children do have more trouble integrating words in a cohesive sentence context. This might arise from the fact that children have less semantic knowledge than adults or from the fact that children have a more limited working memory capacity than adults [95, 96], given that Just and Carpenter [97] relate capacity of the working memory to text comprehension and semantic integration.

Looking at the sizes of the differences between L1 and L2 reading (Table 3), these are subtler and smaller than those found in the comparison between children and adults, except for average fixation duration. We explain this by the fact that our participants have already acquired the skills needed for efficient reading of an alphabetic language (their L1), despite the fact that our participants were not balanced bilinguals, and were clearly less proficient in their L2 (Table 1).

Another similarity between the L2 and the children’s eye movement pattern is the fact that in our dataset the effect of word length on average fixation duration only exists in L2. Studies show a larger word length effect on timed eye movement measures for children compared to adults [6, 73, 76]. This suggests that both children and L2 readers need additional processing time for long words and are thus less efficient at lexical processing [77].

#### Compatibility of results within E-Z reader model

We will argue in the following paragraphs that all of the changes discussed above have one and the same underlying cause, which can be easily accounted for by the E-Z reader model.

The first cause of the longer reading times is the rise in the number of fixations when bilinguals read in L2. This is in part due to less skips and more re-fixations of words. Following the rationale of the E-Z reader model, when the eyes land in a word, the programming of an intra-word saccade is immediately initiated. When this programming is faster than the familiarity check of the fixated word, the intra-word fixation is made [98]. The higher fixation count in L2 reading can thus be related to a slower familiarity check, the first phase of lexical access.

The second reason for the slower reading speed is that the average fixation duration is longer for L2 reading compared to L1 reading. This difference is rather considerable (on average ± 20ms) and can also be related to a slower lexical processing for L2 reading. If more time is needed to identify a word in L2, the eyes should rest longer at the same location. This is exactly what we found.

The third one is that skipping of words is more rare when reading in L2. When the familiarity check of a parafoveal word is completed before the saccade programming to that word is completed, the E-Z reader model predicts that this word will be skipped [98]. More words are skipped in L1 than in L2. This probably means that the familiarity check can be completed faster when reading in the mother tongue than when reading in L2. It follows from the differences in skipping rate that when reading in L2, participants made smaller saccades compared to reading in L1 and monolinguals.

The differences between L1 and L2 reading concerning reading time, saccade length and average fixation duration are inflated in long sentences. This indicates that sentences with more words pose an extra burden on L2 language processing. This might be caused by the fact that longer sentences tend to be syntactically more complex and will have more clauses than short sentences. This will cause larger jumps from one part of the sentence to the next and longer fixation durations because of longer semantic integration times.

In conclusion, all of these findings are consistent with a more effortful familiarity check and slower overall lexical processing for bilinguals reading in L2. Considering that the familiarity check is dependent on word frequency, which is off course subjectively lower for L2 (weaker links), and predictability, the bilingual L2 disadvantage in visual language processing might be reduced to a quantitative difference of exposure to the lexical items in the lexicon. Reichle et al. [31] already showed that the eye movement pattern of children could be modeled by simply reducing the rate of lexical processing. Given that we established a close parallel between patterns of eye movement in children and L2 readers, we hypothesize the same, although smaller, adjustment to the E-Z reader model parameters could possibly also model the L2 reading pattern of unbalanced bilinguals.

### Bilingual L1 vs. Monolingual Reading

The weaker links account predicts a drop in the strength of the links between all word forms and their representations in the bilingual lexicon because reading practice is divided across more (almost double the amount of) lexical items [67]. Therefore, this account predicts slower silent reading for bilinguals. Although some studies [71, 72] do report such a bilingual disadvantage for isolated word recognition, this was never investigated for language comprehension in a natural reading context when the target language proficiency was matched across the bilingual and monolingual group.

Contrary to predictions made by the weaker links account, we did not find a clear general disadvantage for bilinguals reading in their mother tongue compared to monolinguals. We did find a small bilingual disadvantage for fixation count per sentence. Bilinguals fixate slightly more often than monolinguals, but only in sentences with more than 23 words. Also, the amount of fixations that bilinguals made is more strongly determined by the average word length of the sentences than it is for monolinguals. Importantly, there is no interaction of word length or number of words with L1 proficiency. This means that these subtle differences are indeed caused by having a second language and not by a possibly reduced L1 language proficiency for bilinguals. Remember that this bilingual disadvantage does not show in the overall sentence reading time, while in production substantial bilingual slowing of reaction times was found [68]. In lexical decision tasks, the evidence is more mixed. Ransdell and Fischler found a significant disadvantage for bilinguals in their first acquired language compared to monolinguals [71]. Duyck et al. did not find any difference in reaction times for Dutch-English bilinguals and English monolinguals [99]. These bilinguals were taken from the same population, as the one tested in the current paper.

We want to point out that our design was very sensitive: we were able to detect significant differences of 0.2 fixations per sentence. This adds robustness to the observed null effect for bilingual L1 and monolingual sentence reading times. On top of that the bilinguals actually show a slightly faster sentence reading time than the monolinguals do (Table 3) although this difference does not reach significance.

Gollan and Acenas assume a reduced integration between semantic and phonological codes in bilingual language production [67]. It is very unlikely that similar weaker links for bilingual comprehension would not have an impact on lexical access and thus on fixation durations and reading times. From this, we could conclude that the weaker links theory does not provide a full picture of the underlying reason for the more subtle bilingual comprehension disadvantage we found in our unbalanced bilingual population with a late age of acquisition of L2.

Gollan et al. also predicted that the bilingual disadvantages would be smaller in comprehension than in production because the latter is less practiced, more difficult and involves more levels of processing where frequency is important [13]. One has to consider that comprehension and production processes might be very distinct. In order to speak in one language (production), the speaker has to by definition make a language selection. In a picture-naming task, the picture needs to be named either in L1 or L2, and one of the two lexical representations needs to be inhibited, during each utterance. Such inhibition is not necessary in reading, in which bilinguals may rely on bottom-up information coming from the visual input to the lexical representation. Even if lexical representations from both languages become active, an actual language selection is not needed, and therefore recognition implies less inhibition than production does. Since some have proposed that distinct lexical forms serve comprehension and production [100, 101] it is not improbable that being a bilingual would have a different impact on the representational strength of the lexical entities in comprehension than on those in production.

Finally, we want to stress that the participants in this study are different than those who are usually used in the studies reporting an L1 disadvantage for bilinguals [64, 72]. It is therefore completely possible that the weaker links account holds for bilinguals who indeed have less exposure to their L1 due to an increased exposure to L2. These are mostly balanced or early bilingual populations. As described in the introduction, we do not think however that the weaker links hypothesis necessarily holds for all bilingual populations. More specifically, late L2 learners, who have acquired full L1 proficiency before acquiring an L2 are likely to have a larger language exposure overall than monolinguals do, due to an active seeking of extra language exposure. Also, late bilinguals are more likely to have already developed a certain level of lexical entrenchment for words in L1, before acquiring the new L2 words. This makes the ‘weakening’ of links between L1 semantic representations and word forms rather unlikely.

In conclusion, our results show no evidence that unbalanced late bilinguals read slower in their L1 than monolinguals do. Any possibly subtler differences (e.g. fixation counts or other differences), which may emerge at a word level, are at least compensated elsewhere so that on a whole, unbalanced bilinguals do not show any disadvantages compared to monolinguals when reading in L1. These findings imply that at least for unbalanced bilinguals, no ‘weaker’ links have to be assumed to understand bilingual language processing. This is compatible with the notion that language comprehension and production might overlap only at the level of meaning [100, 101], or at least are not completely shared or aligned [13].

#### Comparison of L1 data with meta-analysis of eye movements

It is important to compare our L1 results with the other eye movement studies that have already reported sentence level measures, in order to establish to what extent such results are generalizable to different settings. When we compare our average reading parameters with a meta-analysis by Rayner [17], mostly including findings from earlier sentence-embedded eye movement research, we observe some slight deviations: We found shorter average fixation durations, longer saccade lengths and a higher regression rate (Table 4).

**Table 4:** A comparison of the reading meta-analysis of Rayner [17], based on sentence reading research, and our natural L1 reading data. In our analysis we use first pass skipping rate (52%), but in the table we report total skipping rate (41.5%).

	Sentence Reading	Book Reading
	Rayner [17]	Our L1 Data
Avg Fixation duration	225-250ms	215.8ms
Avg Saccade length	7–9 characters	9.9 characters
Regression Rate	10%-15%	24.2%
Fixations per 100 words	75–118	72
Skipping probability		
Content words	15%	34.2%
Function words	65%	48.8%

These differences indicate that reading a continuous text or story is not the same as reading isolated sentences [91]. Radach et al. found that the overall fixation duration of words is longer for reading passages, but the first pass measures are slower than when reading isolated sentences [91]. Radach et al. explain this by suggesting that readers of passages of text perform a fast first pass across the text followed by a rereading of the passage. This is compatible with our findings, illustrated by lower average fixation durations, longer saccade lengths and more regressions compared to results from isolated or sentence embedded research (Table 4). Additional evidence for this difference in reading strategy comes from analyses of natural reading data, that found regression rates (21% for adults, 36% for 10–11 year olds) and fixation durations (200ms for adults, 243ms for 10–11 year olds) that are similar to ours [102]. As inter-word regressions indicate integration difficulties, it is plausible that people reading individual sentences would have less need to move their eyes back in the text. This is compatible with Radach et al., who state that when reading continuous text, people reread the text after a fast first pass [91].

### Limited Effects of Proficiency and Word Frequency

Our data suggests that the influence of proficiency on sentence level reading parameters is small. In fact, we only find a significant effect of L2 proficiency in the fixation count analysis of the L1 vs. L2 reading comparison. The sentence-level differences in eye movements between L1 and L2 reading are apparently not very sensitive to the L2 proficiency level of the bilinguals. Our bilingual participants were all L1-dominant, unbalanced bilinguals who nevertheless showed considerable variation in L2 composite proficiency scores [52.5%-86.8%]. Note that this range was large enough to yield an effect of L2 proficiency for fixation count. A 10-point increase in the L2 composite proficiency score yields about a decrease of 1.35 fixations per 100 L2 words. For example, a person scoring 65% on his/her L2 proficiency would fixate 92.9 times per 100 words, a person scoring 75% would then on average make 91.6 fixations per 100 words. When we look at the fitted value of the least L2 proficient bilingual scoring lowest on L2 we observe 94 fixations per 100 words, while the highest L2 proficient person has 90 fixations per sentence. When we use the L2 LexTALE scores instead of the L2 composite proficiency score we observe even smaller effects. Here, a 10-point increase in the L2 LexTALE score yields about a decrease of 0.51 fixations per 100 words. A person scoring 65% on his/her L2 proficiency would fixate 76.8 times per 100 words, and a person scoring 75% would make 76.3 fixations per 100 words. The difference between the highest scoring bilingual on the L2 LexTALE and the lowest scoring bilingual is only 0.3 fixations. Even though these effects are small, they are nevertheless detected, illustrating that proficiency just does not yield big effects on sentence reading measures, rather than that these null interaction effects are caused by a small range of L2 proficiency scores for the tested bilinguals. Our results suggest that the differences in eye movement pattern between L1 and L2 reading are more determined by the fact that the L2 is acquired after the L1, than merely by the L2 language proficiency. The absence of (strong) L2 proficiency interactions effects also supports the generalizability of these findings to other unbalanced bilingual populations with somewhat different L2 proficiency scores. Of course, for balanced bilinguals, a different pattern may emerge.

In our analyses, we find few effects of or interactions with word frequency. This is not surprising, given that word frequency measures affect early measures of language processing, like single fixation durations and first fixation durations [103] and have a smaller effect on natural reading than on reading of isolated words or sentence embedded target words [57, 91]. The low frequent words would be more easy to process in continuous text because of the context it provides to identify such a word [57]. An additional reason for the absence of an influence of frequency is that the focus of this paper was on sentence reading parameters, and therefore we used an average frequency measure of only the content words in the sentence. This is likely to be a rather insensitive measure, and any frequency effects may be compensated by words on the other end of the scale. This hypothesis is confirmed in a separate study, where we have analyzed frequency effects in word-level eye movements of this corpus [79]. Here we found clear effects of word frequency in L1, L2 and monolingual reading.

Another issue in our analyses is that we find some 3-way interactions, with rather small effect sizes, that we did not predict or expect. They may offer inspiration for future research that is aimed at more specific questions than the current paper, using smaller, controlled experiments, aimed specifically at that interaction effect.

### Further use of Parameters/Findings

From our analyses, it is clear that the eye movement behavior of bilinguals in L2 shows some similarities to the reading behavior of children. The most parsimonious explanation is that both patterns have the same underlying cause: slower lexical processing. This suggests that the L2 reading pattern could possibly be modeled in the E-Z reader model by changing the same parameter as Reichle et al. used in modeling the reading pattern of children [31]. It would be interesting for further research to try and simulate the results of this corpus with the E-Z reader model.

The same pattern of changes from adult to child reading has been consistently found in German, Finnish and English. This is remarkable because English and Finnish are dissimilar languages [104]. If we draw our parallel even further, we might then assume that the differences that we found between L1 and L2 reading will be universal and consistent across different language pairs, given that the bilingual participants acquired L2 later than their L1 and are less proficient in their L2 than they are in their L1. There is additional evidence that these results will generalize to bilingual populations with other L1 languages than Dutch [44]. It has been shown that although cross-language influences, like cognate status of words, exist, word recognition by bilinguals in L2 is mostly determined by within language factors, like frequency and word length [44].

## Conclusion

In summary, we have analyzed the sentence level eye movement behavior of bilinguals reading in L1 and L2, and of monolinguals reading in their mother tongue. We find large differences between sentence reading in the dominant language (L1) and a later acquired language (L2). All of these differences can be paralleled to the reading pattern found in 7–11 year old children acquiring reading skills, although the differences between L1 and L2 reading are smaller than the differences between adult’s and children’s reading pattern. These changes are all compatible with the concept of a general slowing of the process of lexical access, in parallel with the modeling effort of Reichle et al. [31].

We do not find clear disadvantages for bilinguals reading in L1 compared to monolinguals reading in their only language. This shows that the bilingual disadvantage found in language production tasks and comprehension tasks using isolated words as stimuli, is not universally present across all modalities of language use or all bilingual language users. This means that the weaker links account [64], which did a good job accounting for the balanced bilingual disadvantage in production, might not apply for comprehension of continuous text or language processing of unbalanced late bilinguals.

We hope these findings will inspire future research targeted at specific effects reported here, and promote the method of eye tracking of natural language reading, parallel to continued isolated word recognition research.

## Supporting Information

S1 File Additional Tables. (PDF)

S2 File English Multiple Choice-Questions. (PDF)
