# Supplementary material for: Eye Movement Patterns in Natural Reading: A Comparison of Monolingual and Bilingual Reading of a Novel
Source: PLoS One. 2015 Aug 19;10(8):e0134008. doi: 10.1371/journal.pone.0134008 (PMC4545791; doi:10.1371/journal.pone.0134008)
Supplement: S1 File — (PDF) [file pone.0134008.s001.pdf]

Additional Tables

Table A

Count of bilingual participants agreeing and not agreeing on second language skills items.

**Skills**	Agree	Don't Agree
Carry on normal conversation in L2	19	0
Watch television shows in L2	19	0
Listen to music in L2	19	0
Read and comprehend questions in L2	19	0
Read books or articles in L2	19	0
No problems in understanding L1 speaker	18	1
Carry on a discussion in L2	17	2
Love speaking L2	16	3
Explain difficult situation in L2	15	4
Answer difficult questions in L2	12	7
Think in L2	11	8
Speak to myself in L2	10	9
Write in L2	8	11
Make no/ almost no mistakes in L2	6	13
Dream in L2	5	14

Table B

Count of bilingual participants agreeing and not agreeing on second language switching items.

Switching	Agree	Don't Agree
I'm sometimes in a tip of the tongue state	16	3
I sometimes can't get the right word	14	5
I use a different language when I do not remember a word	13	6
I often use different languages intermixed	9	10
I often use different languages intermixed without noticing	5	14
I sometimes speak in a language that my dialogue partner doesn't understand	5	14
