# Supplementary material for: Eye Movement Patterns in Natural Reading: A Comparison of Monolingual and Bilingual Reading of a Novel
Source: PLoS One. 2015 Aug 19;10(8):e0134008. doi: 10.1371/journal.pone.0134008 (PMC4545791; doi:10.1371/journal.pone.0134008)
Supplement: S2 File — (PDF) [file pone.0134008.s002.pdf]

English multiple-choice questions with possible answers that were used to test text comprehension. The correct answer is indicated in bold.

1. Who does Mr. Hastings meet for the first time in the front garden when Mr. Hastings arrives at Styles Court?

a) John Cavendish

b) Mary Cavendish

c) Evelyne Howard

d) Emily Inglethorpe

2. The book is narrated by Mr. Hastings. Of what secret ambition does he tell Mary Cavendish?

a) Becoming a biographer.

b) Becoming a police officer.

c) Becoming a detective.

d) Becoming a doctor in medicine .

3. After Evelyne Howard leaves Styles Court because of a discussion with Emily Inglethorpe she goes to live in Middlingham. What position does she find there?

a) nanny

b) housekeeper

c) nurse

d) pharmacist

4. What is the nickname of the colleague of Cynthia Murdoch?

a) Nibs

b) Buns

c) Barny

d) Snug

5. Who wakes Mr. Hastings on the night of the murder?

a) Nobody. He wakes up because of the noises.

b) Lawrence Cavendish.

c) John Cavendish.

d) Dorcas.

6. How does Alfred Inglethorpe explain that he did not sleep in his bed on the night of the murder?

a) **He forgot his house key.**

b) He didn't want to wake his wife.

c) Friends of his needed his help.

d) He was drunk and slept with friends.

7. Why is the fact whether Emily Inglethorpe ate on the evening of her death of importance for the investigation?

a) Her appetite says a lot about her emotional state.

b) A big dinner could have slowed down the effect of the poison.

c) A big dinner could have aggravated the effect of the poison.

d) It is not important to the investigation.

8. Which of the following statements is not correct? When Poirot investigates the bedroom of Emily Inglethorp for the first time he

a) notices a coffee cup stamped to pieces.

b) discovers a wet spot on the carpet.

c) takes the documents out of the purple case.

d) discovers the remains of a will in the ashes of the fireplace.

9. The sixth point of importance is

a) a piece of green fabric Poirot finds.

b) That Dorcas overheared the argument between Mrs. and Mr. Inglethorpe

c) the spare key to the purple box was missing

d). An empty box of sleeping powders

10. What did Annie notice was strange about the cocoa?

a) It was not heated yet.

b) there was some kitchen salt on the tray.

c) the tray was displaced by somebody else.

d) the cocoa was brought up later than usual.

11. How many coffee cups does Poirot count in the drawing room?

a) 3

b) 5

c) 6

d) 7

12. What is true about the last intact will of Mrs. Inglethorpe?

a) She left her entire fortune to John Cavendish.

b) She left her entire fortune to Alfred Inglethorp.

c) She left her entire fortune to Evelyne Howard.

d) She left her entire fortune to Lawrence Cavendish.

13. What does Lawrence think is the cause of dead of his stepmother?

a) She was poisoned.

b) Tetanus.

c) Heart failure.

d) Old age.

14. Who admits to not being a good friend to Mr. Hastings?

a) John Cavendish.

b) Hercule Poirot.

c) Lawrence Cavendish.

d) Mary Cavendish.

15. What element does Poirot thinks is implicating Alfred Inglethorpe in the murder?

a) that he was not in the house at the moment of the murder.

b) that the door of Mrs. Inglethorpes bedroom was closed from the inside so she must have opened the door for her husband.

c) that everybody else suspects him.

d) that Alfred Inglethorpe had an argument with Emily Inglethorpe on the day of her murder.

16. Two people are observed to shed tears for the murdered Emily Inglethorpe. Who are they?

a) Evelyne Howard and Hercule Poirot

b) Evelyne Howard and Dorcas

c) Evelyne Howard and Mary Cavendish

d) Evelyne Howard and Cynthia

17. Why is it impossible according to Dr. Wilkes that Emily Inglethorpe was poisoned by her medicine?

a) Because it did not contain any poisonous substances.

b) Because Emily Inglethorpe was too smart to take an overdose.

c) Because the dose of strychnine in the medicine is too small to poison somebody.

d) Because Emily Inglethorpe did not take the medicine that night.

18. How does Alfred Inglethorpe explain the last words of his wife?

a) She was accusing him of her death.

b) She was trying to tell him something.

c) She wrongfully thought that Dr. Bauerstein was Mr. Inglethorpe.

d) She was talking nonsense.

19. What two things are of primary significance to Poirot about the day of the murder?

a) The temperature and the green fabric he found.

b) The temperature and the distinctive appearance of Alfred Inglethorpe.

c) The temperature and the broken cup

d) The broken cup and the distinctive appearance of Inglethorpe

20. Why is Poirot determined that Alfred Inglethorpe shall not be arrested for the murder of Emily Inglethorpe?

a) Alfred will be found innocent.

b) Alfred is innocent.

c) There is not enough evidence to arrest Mr. Inglethorpe.

d) The real murderer will escape.

21. What is the reason that Alfred Inglethorp could not give a believable alibi for the time the strychnine was bought in the pharmacy?

a) He was scared to admit he had been with Mrs. Raikes.

b) He does not have an alibi.

c) He was taking a walk and nobody could confirm this story.

d) He wanted to be arrested.

22. Who inherits ‘Styles Court’?

a) Alfred Inglethorpe.

b) John Cavendish.

c) Lawrence Cavendish.

d) Nobody.

23. Which statement is true?

a) Annie shows Poirot where to find the dressing-up trunk

b) Poirot finds a green dress and a fake beard in the dressing-up trunk.

c) Poirot finds a green dress in the dressing-up trunk.

d) Poirot finds a fake beard when looking for a green dress.

24. Which person does Evelyne Howard's intuition tells her commited the murder?

a) Cynthia Murdoch

b) Alfred Inglethorp

c) She does not say who.

d) Mr. Hastings

25. Where does Mr. Hastings hear Mary and John Cavendish arguing?

a) the park

b) their bedroom

c) in the village

d) in the drawing room

26. When Cynthia Murdoch confides in Mr. Hastings, she tells him that certain people in the household hate her. Who is she talking about?

a) John and Lawrence Cavendish

b) Emily Howard en Mary Cavendish

c) Lawrence Cavendish and Emily Howard

d) Lawrence and Mary Cavendish

27. The mysterious Dr. Bauerstein, although he does not turn out to be the murderer, nonetheless does turn out to be a criminal. What is his crime?

a) Espionage

b) Burglary

c) Embezzlement

d) Blackmail

28. Which of the following is true of Mary Cavendish?

a) Her first husband died in prison.

b) Her father died under mysterious circumstances.

c) Her mother was Russian.

d) Her father was shot for being a traitor.

29. The fourth letter that Mrs. Inglethorpe had sent on the day before the murder was addressed to…

a) Evelyne Howard.

b) Alfred Inglethorp.

c) A French music publisher.

d) A nurse in Middlingham.

30. Whose fingerprints were found on the bottle of strychnine in the pharmacy?

a) Lawrence Cavendish.

b) Lawrence Cavendish and Cynthia Murdoch.

c) Lawrence Cavendish and John Cavendish.

d) Cynthia Murdoch.

31. In the case against John Cavendish, what contention of the prosecution will Poirot be able to refute?

a) That it was John who bought the strychnine in the pharmacy.

b) That John would benefit from the death of Emily Inglethorpe.

c) That John brought the coffee to Mrs. Inglethorpe's room.

d) **That John was the one who burned the will.**

32. In the court case against John Cavendish, whom else is the prosecution trying to implicate in the murder?

a) Mary

b) Evelyne

c) **Lawrence**

d) Hastings

33. How does the defense explain the bottle of poison in John Cavendish's room?

a) Somebody is trying to frame John Cavendish

b) He used this to poison a stray dog.

c) John hid this bottle for somebody else he is trying to protect.

d) There was no bottle of poison in John's room.

34. Which of the following statements is false?

a) John Cavendish claims that he does not remember the exact words that were used by his step mother in their discussion.

b) The prosecution believes that John bought the strychnine in the pharmacy.

c) There is a consensus in the court room that the handwriting on the poison list in the pharmacy is not Alfred Inglethorp's.

d) Lawrence denies that he touched the bottle of strychnine in the pharmacy.

35. In addition to the strychnine, Emily Inglethorpe (along with Cynthia) was given a mild narcotic. Who drugged Emily Inglethorpe and Cynthia Murdoch?

a) Dr. Bauerstein

b) Mary Cavendish

c) John Cavendish

d) Evelyne Howard

36. How was Emily Inglethorpe poisoned?

a) Bromide was added to her medication.

b) Strychnine was added to her coffee.

c) Strychnine was added to the bromide powders.

d) Strychnine was added to her medication.

37. Which of the following is true of Evelyn Howard and Alfred Inglethorp's relationship?

a) They hate each other intensely.

b) They are trying to frame each other for murder.

c) They suspect each other of committing the murder.

d) They are cousins.

38. Whom does Evelyn Howard attempt to implicate in the murder?

a) Mary Cavendish

b) Cynthia Murdoch

c) Alfred Inglethorpe

d) John Cavendish

39. How does Poirot realize that there is a letter in the vase?

a) There was one vase more on the mantle the first time he entered the room.

b) The vases were empty first.

c) He had to straighten the objects on the mantle twice.

d) The objects on the mantle were straight while they were crooked first.

40. Why did Poirot want John Cavendish to go on trial for the murder of Emily Inglethorpe?

a) He believed John was guilty.

b) John was interfering with the investigation.

c) He believed it would cause the real killer to confess.

d) He thought it would bring him and his wife closer together.
